# Prognostic Value of Perineural Invasion in Oral Tongue Squamous Cell Carcinoma: A Systematic Review and Meta-Analysis

**DOI:** 10.3389/fonc.2021.683825

**Published:** 2021-07-12

**Authors:** Jiajia Li, Shan Liu, Zhangao Li, Xinxin Han, Lin Que

**Affiliations:** ^1^ State Key Laboratory of Oral Diseases and National Clinical Research Center for Oral Diseases, West China Hospital of Stomatology, Sichuan University, Chengdu, China; ^2^ Department of Pediatric Dentistry, School of Stomatology, The Fourth Military Medical University, Xi’an, China

**Keywords:** head and neck cancer, oral tongue squamous cell carcinoma, perineural invasion, survival, meta-analysis, locoregional recurrence

## Abstract

**Objectives:**

A significant number of recently published research has outlined the contribution of perineural invasion (PNI) to clinical outcomes in oral tongue squamous cell carcinoma (OTSCC), but some results remain conflicting. This study aimed to determine whether patients with OTSCC with PNI have a worse prognosis than those without PNI.

**Materials and Methods:**

PubMed, Embase, and the Cochrane Library were queried for potentially eligible articles published up to December 2020. The primary outcomes were the hazard ratio (HR) for locoregional recurrence, overall survival (OS), disease-free survival (DFS), and cancer-specific survival (CSS). The random-effect model was used in all analyses.

**Results:**

Seventeen studies (4445 patients) were included. Using adjusted HRs, the presence of PNI was associated with a higher risk of locoregional recurrence (HR=1.73, 95%CI: 1.07-2.79, P=0.025, I^2^ = 33.1%, P_heterogeneity_=0.224), worse OS (HR=1.94, 95%CI: 1.39-2.72, P<0.001, I^2^ = 0.0%, P_heterogeneity_=0.838), worse DFS (HR=2.13, 95%CI: 1.53-2.96, P<0.001, I^2^ = 48.4%, P_heterogeneity_=0.071), and worse CSS (HR=1.93, 95%CI: 1.40-2.65, P<0.001, I^2^ = 25.5%, P_heterogeneity_=0.251). PNI had an impact on locoregional recurrence in early-stage OTSCC but not in all stages, and on OS, DFS, and CSS in all-stage and early-stage OTSCC. The sensitivity analyses showed that the results were robust.

**Conclusion:**

The presence of PNI significantly affects the locoregional recurrence and survival outcomes among patients with OTSCC.

## Introduction

Oral squamous cell carcinoma (OSCC) is the sixth most common type of cancer worldwide (1, 2), and, among patients with OSCC, oral tongue squamous cell carcinoma (OTSCC) has been reported as the most common cancer found in the oral cavity (3, 4). The prognosis of OSCC is generally poor (1, 2, 5), and the prognosis of OTSCC is even poorer (6, 7).

Perineural invasion (PNI) is the result of a complex interaction between invading tumor cells and the particular perineural niche, which has been noted to affect outcomes in many cancers (8–11). PNI is observed in 70%-100% of pancreatic cancers, 7%-76% of gastric cancers, 56%-88% of biliary tract tumors, 12%-84% of prostate cancers, 5%-90% of head and neck cancers, 16%-39% of colorectal cancers, and 9%-31% of cervical cancers (8). In all these cancers, PNI is independently associated with poorer prognosis and shorter survival, except for prostate cancer, in which PNI is associated with clinical locoregional recurrence (8). In head and neck cancer, PNI is typically defined by tumor cells invading perineural tissues, tracking along nerves, and/or surrounding at least one-third of the circumference of nerves, and has been defined by the work of Leibig et al. (12), among others (13). In oral cancer, PNI is a high-risk factor for poor outcomes (14). PNI in oral cancer is an indication for radiotherapy and systemic therapy (5, 15–18). PNI is a significant predictor of poor outcomes and a requirement for adjuvant therapy in head and neck squamous cell carcinoma (HNSCC) (19–21). PNI is associated with the depth of invasion of OTSCC (22) and cervical lymph node involvement and might suggest consideration for elective neck dissection (23), even in stage 1 and 2 diseases (24). A significant number of recently published research has outlined the contribution of PNI to clinical outcomes in OTSCC (10, 19–21, 25–34). Some authors advocate incorporating PNI in the staging systems for OTSCC (34). Nevertheless, some results remain conflicting.

PNI is usually described in pathological reports, and it may influence the therapeutic management of the patients (12, 13). Therefore, this systematic review and meta-analysis aimed to determine whether patients with OTSCC with PNI have a worse prognosis than those without PNI.

## Materials and Methods

### Literature Search

This systematic review and meta-analysis was performed according to the Preferred Reporting Items for Systematic Reviews and Meta-Analyses (PRISMA) guidelines (35). PubMed, Embase, and the Cochrane Library were queried for potentially eligible articles published up to December 2020, based on the PICO principle (36) and screening according to the eligibility criteria: 1) population: patients with OTSCC; 2) exposure: PNI; 3) outcome: prognostic outcome related to survival with a hazard ratio (HR) reported in a Cox regression model; and 4) language limited to English. **Supplementary Table S1** presents the search terms. Multiple studies using the same group of patients were considered duplicated; only the most recent article meeting the eligibility criteria was included.

### Data Extraction

Study characteristics (authors, year of publication, country, study design, sample size, and sex and age of the patients), exposure-related parameters (OTSCC stage and the TMN staging version), and the primary outcome [HR for locoregional recurrence, overall survival (OS), disease-free survival (DFS), and cancer-specific survival (CSS)] were extracted by two investigators (Jiajia Li and Shan Liu) according to a pre-specified protocol. Locoregional recurrence included local and regional recurrence and DFS encompasses all aspects of the disease (local, regional, and distant). Discrepancies were solved by the discussion by referring to the original paper.

### Data Synthesis

The HRs and their confidence intervals (CIs) that indicated the prognostic outcome were extracted to summarize the prognostic effects of PNI. Whenever the HRs were reported using univariable and multivariable models, the HRs were extracted from the multivariable model with the most covariables.

### Quality of the Evidence

The search ultimately yielded one randomized controlled study (RCT), three prospective cohort studies, and 13 retrospective studies. The level of evidence of RCT and cohort studies was assessed according to the Cochrane Handbook (37), and the Newcastle-Ottawa Scale (NOS) criteria (38), respectively, and Methodological Items for Non-Randomized Studies (MINORS) (39) were used to assess the other studies. Quality assessments were completed independently by two authors (Zhangao Li and Lin Que). Discrepancies in the assessment were resolved through discussion until a consensus was reached. The GRADE method was used to determine the degree of certainty of the outcomes (37, 40).

### Statistical Analysis

All analyses were performed using STATA SE 14.0 (StataCorp, College Station, TX, USA). Effects and corresponding 95% CIs were used to compare the outcomes. Analyses were done separately for the adjusted and unadjusted HRs. Statistical heterogeneity among studies was calculated using Cochran’s Q-test and the I^2^ index. An I^2^ >50% and Q-test P-value <0.10 indicated high heterogeneity. The random-effects model was used in all analyses, irrespective of heterogeneity, to account for differences among study populations, treatment regimens, and local practices (37, 41). The publication bias was not assessed because each meta-analysis included <10 studies, in which case funnel plots and the Egger and Begg tests could yield misleading results (37, 42). P-values <0.05 were considered statistically significant.

## Results

### Selection of the Studies

**Figure 1:**
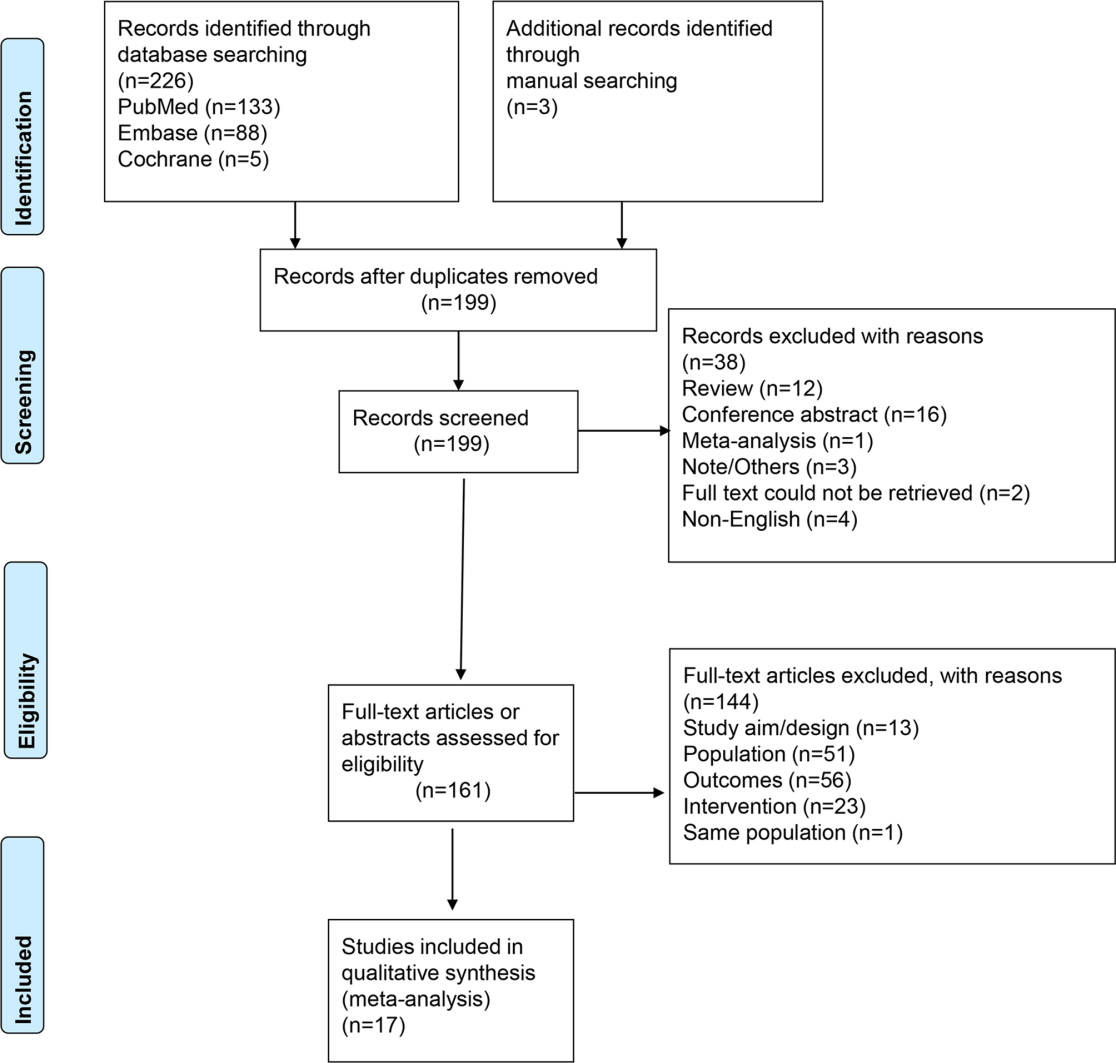
PRISMA 2020 flow diagram.

**Figure 1** presents the study selection process. The initial search yielded 229 records; after removing the duplicates, 199 were screened, and 38 were excluded. Then, 161 full-text articles or abstracts were assessed for eligibility, and 144 were excluded: 13 for study design/aim, 51 for the population, 56 for the outcomes, 23 for the intervention, and one for the language. Finally, 17 studies were included in this meta-analysis. **Supplementary Table S2** presents the excluded studies.

**Table 1** presents the characteristics of the studies (20, 25–32, 43–50), including one RCT (43), three prospective cohort studies (44–46), and 13 retrospective studies (20, 25–32, 47–50). Eight studies were from Asia (20, 30, 32, 43, 44, 46, 47, 50), one was Australia (45), three were from Europe (25, 28, 29), and five were from North America (26, 27, 31, 48, 49). The 17 studies included a total of 4445 patients. **Supplementary Table S1** shows that the RCT (43) had a low risk of bias. Among the cohort studies, three (26, 30, 49) scored 7 stars, four (27, 29, 47, 48) scored 8 stars, and two (44, 45) scored 9 stars (**Supplementary Table S1**). For the other studies evaluated using MINORS, one (32) scored 5 points, five (20, 25, 28, 31, 50) scored 6 points, and one (46) scored 8 points (**Supplementary Table S3**).

**Table 1:** Literature search and study characteristics.

Author, year	Country	Study design	Sample size	Stage	TNM staging guideline	Male, %	Age	Uni/multivariate analysis	Adjusted factors	Main results
Yang, 2018 (43)	China	RCT	221	cT1-T2N0	AJCC7	NA	NA	Multivariate	T stage, Pathological grade	LN, metastasis, LR, DFS
Choi, 2017 (44)	Korea	Prospective cohort	75	cT1-T2N0	NA	56	52.1 (49.0-55.2)	Uni/multivariate	/	LRSF, RRSF
Subramaniam, 2020 (45)	Australia	Prospective cohort	425	All	AJCC7	71.8	45 (18-86)	Multivariate	/	OS, DSS, Recurrent
Xu, 2020 (46)	China	Prospective cohort	120	cT1-T2N0	AJCC8	75.8	60 (31-75)	Univariate	/	Occult neck lymphnode metastasis and LRC
Almangush, 2015 (25)	Finland	Retrospective	479	cT1-T2N0	NA	54.7	NA	Univariate	/	LRR, OS, CSS
Cracchiolo, 2018 (26)	USA	Retrospective	381	All	NA	58.3	57 (18-96)	Uni/multivariate	Tumor size, adjuvant therapy, and lymph node status	DSS, LRFS, RRFS, DRFS
De Paz, 2019 (47)	China	Retrospective	259	All	AJCC8	86.1	≤51.5(47.5%)	Multivariate	Age and sex	OS, DFS
Durr, 2013 (27)	USA	Retrospective	120	All	AJCC7	55	57.5 ± 15.3	Multivariate	Age, sex, race, Smoking status, Charlson comorbidity score, Overall stage, treatment characteristics	Recurrence, OS, RFS
Goodman, 2009 (48)	USA	Retrospective	339	All	NA	57.5	NA	Multivariate	Study site	OS
Ling, 2013 (20)	China	Retrospective	210	All	AJCC6	53.8	55 (22-88)	Uni/multivariate	/	DSS, OS
Marra, 2019 (28)	Italy	Retrospective	106	All	AJCC7	64.1	61 (51-69)	Multivariate	/	DFS
Mascitti, 2020 (29)	Italy	Retrospective	139	All	AJCC8	61.9	NA	Uni/multivariate	TSR, Stage, WHO Grade, Age, and Gender	DSS, DFS, OS
Ong, 2018 (30)	China	Retrospective	166	pT2	NA	56	52.7 (24-70)	Uni/multivariate	/	LR, DFS, OS
Peng, 2014 (31)	Canada	Retrospective	123	cT1N0	AJCC8	52	56 (27-92)	Univariate	/	LRC
Sharma, 2019 (32)	India	Retrospective	202	All	AJCC8	75.7	54.2 ± 14.2	Uni/multivariate	Age, sex, addictions (tobacco smoking, tobacco chewing and/or alcohol consumption), tumor grade, pathological T (pT) stage, pathological N (pN) stage, PNI, LVI, resection margin status, DOI, and ECE	LRR, OS
Sridharan, 2019 (49)	USA	Retrospective	494	cT1-T3N0	AJCC8	55	59 (23-88)	Univariate	/	LR, LRR
Thiagarajan, 2014 (50)	India	Retrospective	586	All	NA	70.9	NA	Uni/multivariate	/	DFS

AJCC, American joint committee on cancer; DFS, Disease-free survival; DSS, disease-specific survival; CSS, Cancer-specific survival; LR, local recurrence; LRC, Locoregional control; LRR, Locoregional recurrence; LVI, lymphovascular invasion; DOI, depth of invasion; ECE, extracapsular extension; LRFS, local recurrence-free survival; RRFS, regional recurrence-free survival; DRFS, distant recurrence-free survival; RFS, recurrence-free survival; /: not available or not applicable.

### Impact of PNI on Locoregional Recurrence and Survival

**Figure 2:**
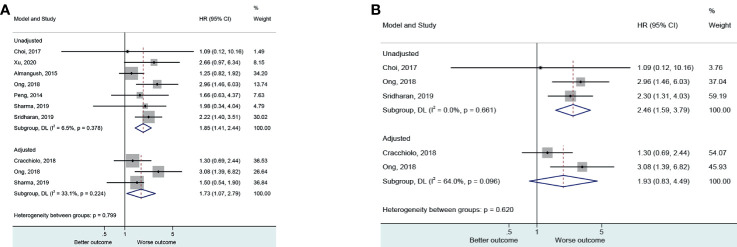
**(A)** Forest plot for locoregional recurrence (local and regional) according to the applied model (adjusted and unadjusted). **(B)** Forest plot local recurrence according to the applied model (adjusted and unadjusted).

Seven (25, 30–32, 44, 46, 49) and three (26, 30, 32) evaluated the non-adjusted and adjusted impact of PNI on locoregional recurrence. The presence of PNI was associated with a higher risk of locoregional recurrence in the non-adjusted (HR=1.85, 95%CI: 1.41-2.44, P<0.001, I^2^ = 6.5%, P_heterogeneity_=0.378) and adjusted (HR=1.73, 95%CI: 1.07-2.79, P=0.025, I^2^ = 33.1%, P_heterogeneity_=0.224) analyses (**Figure 2A** and **Table 2**).

**Table 2:** Hazard ratios for the survival outcome.

Outcome	Model	Subgroup	N (Study)	N (participants)	HR (95%CI)	P	I^2^ (%)	P_heterogeneity_
Recurrence								
	Unadjusted							
		Early	6	1457	1.884 (1.369-2.592)	<0.001	21.9	0.269
		All stage	1	202	1.982 (0.579-6.789)	0.276	\	\
		Prospective	2	195	2.332 (0.984-5.525)	0.054	0	0.47
		Retrospective	5	1464	1.849 (1.315-2.598)	<0.001	28.1	0.234
		Overall	7	1659	1.854 (1.412-2.435)	<0.001	6.5	0.378
	Adjusted							
		Early	1	166	3.082 (1.392-6.824)	0.006	\	\
		All stage	2	583	1.399 (0.896-2.183)	0.139	0	0.748
		Retrospective	3	749	1.726 (1.070-2.786)	0.025	33.1	0.224
		Overall	3	749	1.726 (1.070-2.786)	0.025	33.1	0.224
OS	Unadjusted							
		Early	2	645	1.92 (0.590-6.246)	0.278	81.5	0.02
		All stage	1	202	1.604 (0.930-2.768)	0.09	\	\
		Overall	3	847	1.654 (0.943-2.902)	0.079	65.5	0.055
	Adjusted							
		Early	1	166	2.667 (1.006-7.078)	0.049	\	\
		All stage	4	928	1.862 (1.300-2.668)	0.001	0	0.807
		Overall	5	1094	1.944 (1.387-2.724)	<0.001	0	0.838
								
DFS	Unadjusted							
		Early	1	166	3.201 (1.670-6.137)	<0.001	\	\
		Retrospective	1	166	3.201 (1.670-6.137)	<0.001	\	\
	Adjusted							
		Early	2	387	2.724 (1.747-4.248)	<0.001	0	0.704
		All stage	5	1210	1.949 (1.271-2.988)	0.002	55.5	0.061
		Prospective	1	221	2.560 (1.480-4.429)	0.001	\	\
		Retrospective	6	1376	2.080 (1.412-3.064)	<0.001	53.1	0.059
		Overall	7	1597	2.128 (1.532-2.955)	<0.001	48.4	0.071
CSS	Unadjusted							
		Early	1	479	0.870 (0.511-1.482)	0.608	\	\
		Prospective	1	479	0.870 (0.511-1.482)	0.608	\	\
	Adjusted							
		Early	1	221	3.080 (1.521-6.236)	0.002	\	\
		All stage	4	1155	1.753 (1.285-2.391)	<0.001	8.3	0.352
		Prospective	2	646	1.853 (0.723-4.750)	0.199	77.6	0.034
		Retrospective	3	730	2.067 (1.450-2.945)	<0.001	0	0.821
		Overall	5	1376	1.927 (1.402-2.650)	<0.001	25.5	0.251

OS, overall survival; DFS, disease-free survival; CSS, cancer-specific survival; /: not available or not applicable.

Three (30, 44, 49) and two (26, 30) evaluated the non-adjusted and adjusted impact of PNI on local locoregional recurrence. The presence of PNI was associated with a higher risk of local locoregional recurrence in the non-adjusted (HR=2.46, 95%CI: 1.59-3.79, P<0.001, I^2^ = 0.0%, P_heterogeneity_=0.661) but not in the adjusted (HR=1.93, 95%CI: 0.83-4.49, P=0.126, I^2^ = 64.0%, P_heterogeneity_=0.096) analyses (**Figure 2B**).

**Figure 3:**
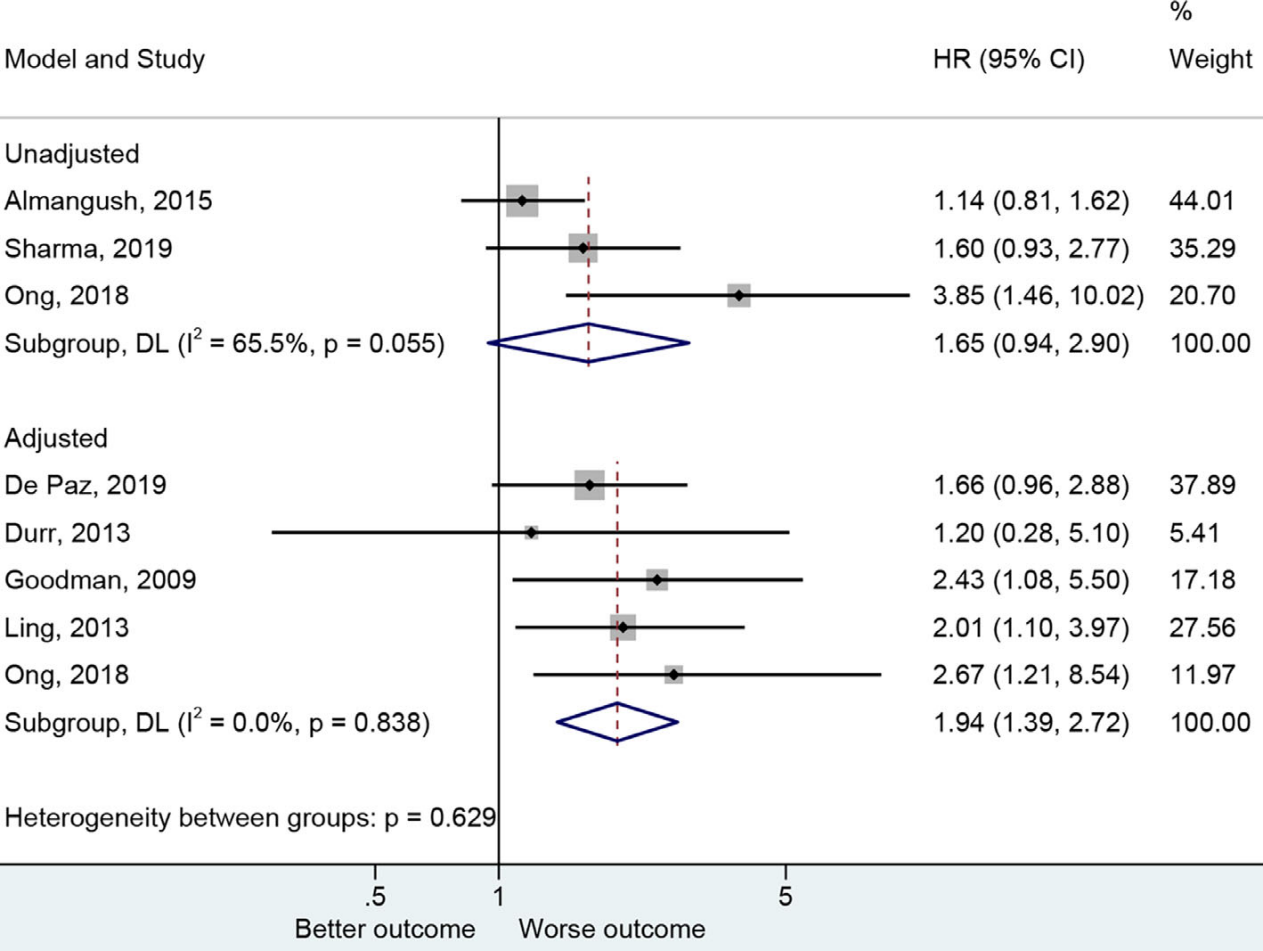
Forest plot for overall survival by the applied model (adjusted and unadjusted).

The presence of PNI was reported with a worse OS (20, 27, 30, 47, 48) in the adjusted analysis (HR=1.94, 95%CI: 1.39-2.72, P<0.001, I^2^ = 0.0%, P_heterogeneity_=0.838) but not in the non-adjusted ones (25, 30, 32) (HR=1.65, 95%CI: 0.94-2.90, P=0.079, I^2^ = 65.5%, P_heterogeneity_=0.055) (**Figure 3** and **Table 2**).

**Figure 4:**
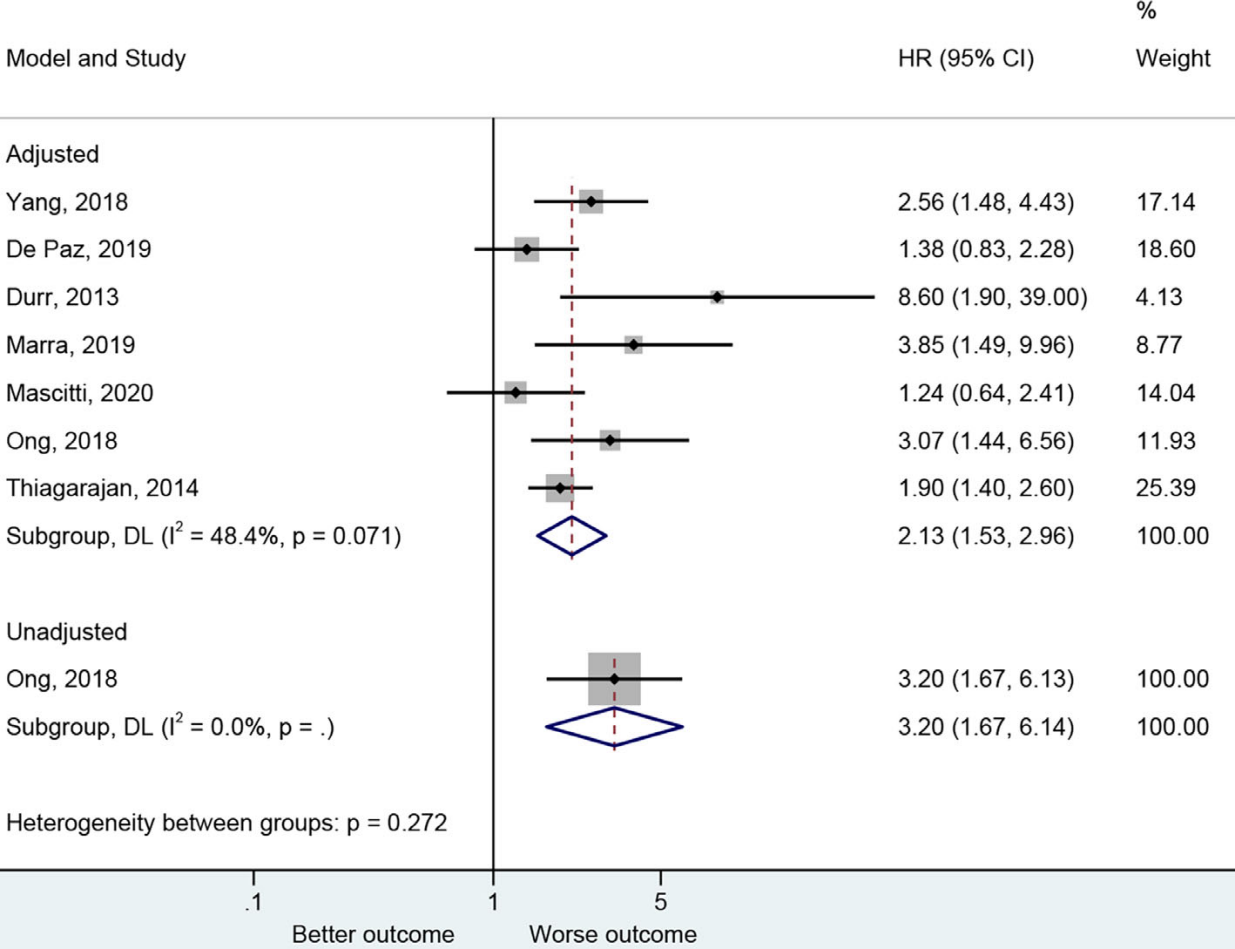
Forest plot for disease-free survival by the applied model (adjusted and unadjusted).

The presence of PNI was reported with a worse DFS in the adjusted (27–30, 43, 47, 50) (HR=2.13, 95%CI: 1.53-2.96, P<0.001, I^2^ = 48.4%, P_heterogeneity_=0.071) and unadjusted (30) (HR=3.20, 95%CI: 1.67-6.14, P<0.001) analyses (**Figure 4** and **Table 2**).

**Figure 5:**
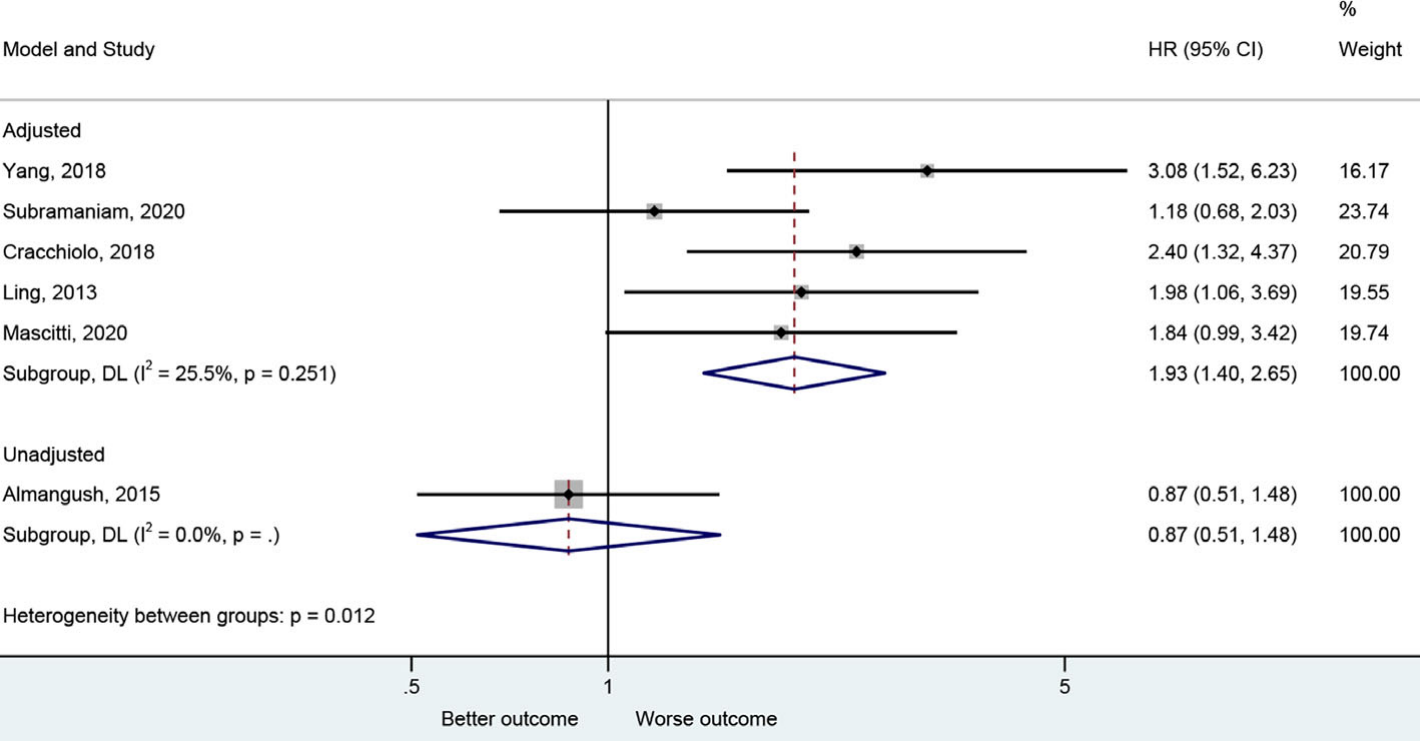
Forest plot for cancer-specific survival by the applied model (adjusted and unadjusted).

The presence of PNI was reported with a worse CSS in the adjusted analysis (20, 26, 29, 43, 45) (HR=1.93, 95%CI: 1.40-2.65, P<0.001, I^2^ = 25.5%, P_heterogeneity_=0.251) but not in the unadjusted one (25) (HR=0.87, 95%CI: 0.51-1.48, P=0.608) (**Figure 5** and **Table 2**).

### Impact of PNI on Locoregional Recurrence and Survival According to Cancer Stage

PNI had an impact on the locoregional recurrence of early-stage OTSCC in the adjusted (30) (HR=3.08, 95%CI: 1.39-6.82, P=0.006) and unadjusted analyses (HR=1.88, 95%CI: 1.37-2.59, P<0.001, I^2^ = 21.9%, P_heterogeneity_=0.269); PNI had no impact on locoregional recurrence in all stages (26, 32) (**Supplementary Figure S1** and **Table 2**). PNI had an impact on the locoregional recurrence of early-stage OTSCC in retrospective studies (25, 30–32, 49) (HR=1.85, 95%CI: 1.32-2.60, P<0.001, I^2^ = 28.1%, P_heterogeneity_=0.234) but not in the prospective ones (44, 46) (**Supplementary Figure S1** and **Table 2**).

PNI had an impact on the OS of early-stage OTSCC in the adjusted (30) (HR=2.67, 95%CI: 1.01-7.08, P=0.049) but not in the unadjusted analyses (25, 30) (HR=1.92, 95%CI: 0.59-6.25, P=0.278, I^2^ = 81.5%, P_heterogeneity_=0.020). PNI had an impact on the OS of all-stage OTSCC in the adjusted (20, 27, 47, 48) (HR=1.86, 95%CI: 1.30-2.67, P=0.001) but not in the unadjusted analyses (32) (HR=1.60, 95%CI: 0.93-2.77, P=0.090) (**Supplementary Figure S2** and **Table 2**).

PNI had an impact on the DFS of early-stage OTSCC (30, 43) (HR=2.72, 95%CI: 1.75-4.25, P<0.001, I^2^ = 0.0%, P_heterogeneity_=0.704) and in all-stage OTSCC (27–29, 47, 50) (HR=1.95, 95%CI: 1.27-2.99, P=0.002, I^2^ = 55.5%, P_heterogeneity_=0.061). Similar results were observed in prospective (43) (HR=2.56, 95%CI: 1.48-4.43, P=0.001) and retrospective (27–30, 47, 50) (HR=2.08, 95%CI: 1.41-3.06, P<0.001, I^2^ = 53.1%, P_heterogeneity_=0.059) studies (**Supplementary Figure S3** and **Table 2**).

PNI had an impact on the CSS of early-stage OTSCC (43) (HR=3.08, 95%CI: 1.52-6.24, P=0.002) and in all-stage OTSCC (20, 26, 29, 45) (HR=1.75, 95%CI: 1.29-2.39, P<0.001, I^2^ = 8.3%, P_heterogeneity_=0.352). Similar results were observed in retrospective (20, 26, 29) (HR=2.07, 95%CI: 1.45-2.985, P<0.001, I^2^ = 0.0%, P_heterogeneity_=0.821) but not in prospective studies (43, 45) (HR=1.85, 95%CI: 0.72-4.75, P=0.199, I^2^ = 77.6%, P_heterogeneity_=0.034) (**Supplementary Figure S4** and **Table 2**).

### Sensitivity Analyses

The sensitivity analyses showed that the sequential exclusion of each study, in turn, did not affect the results (**Supplementary Figure S5**). The GRADE analysis suggests that the degree of certainty is high for all four outcomes (**Supplementary Table S4**).

## Discussion

A significant number of recently published research has outlined the contribution of PNI to clinical outcome in OTSCC (10, 19–21, 25–32), but the results remain conflicting. Therefore, this meta-analysis aimed to determine whether patients with OTSCC with PNI have a worse prognosis than those without PNI. The results indicate that the presence of PNI significantly affects the locoregional recurrence and survival outcomes among patients with OTSCC.

PNI results from the complex interaction between invading tumor cells and the particular perineural niche (8–11). PNI is defined by tumor cells invading perineural tissues, tracking along nerves; since nerves travels across a wide number of structures in the head and neck area, the tumor cells can invade a large area (12, 13). The currently proposed mechanisms suggest that the perineural microenvironment is favorable to tumor cell growth and mobility. Indeed, the nerve microenvironment includes blood supply and numerous cell types that maintain and support the surrounding neurons, but that can also maintain and support tumor cells (51, 52). Several chemokines might also be involved, but their involvement might vary according to cancer type (53, 54).

PNI has been reported to be associated with cancer outcomes in various types of cancer (8–11), including HNSCC (19–21) and OTSCC (20, 27, 28, 30, 43, 48, 50), but the findings in OTSCC are not unanimous (25, 26, 31, 32, 44, 46). Nevertheless, when synthesized using the meta-analysis methodology, these conflicting studies support that PNI is associated with locoregional recurrence and poor OS, DFS, and CSS. This is supported by a previous meta-analysis of PNI in HNSCC, in which PNI was associated with OS, DFS, and CSS (55). The present study refines the results of the previous meta-analysis by showing that the associations remain true in OTSCC, which is a particularly aggressive subtype of HNSCC (5, 27). In head and neck adenoid cystic carcinoma, PNI is independently associated with a poor prognosis, according to a meta-analysis by Ju et al. (56), and the prognosis of PNI was worst in males and young patients, but less definitive results were found by other systematic reviews (57, 58). A previous meta-analysis indicated that PNI is a strong factor predicting local recurrence and survival in colorectal cancer and that the prognostic value of PNI was similar to that of the depth of invasion, tumor differentiation, positive lymph nodes, and lymphatic and extramural invasion (9). In invasive cervical carcinoma, PNI is associated with OS but not with DFS (59). Previous meta-analyses also reported similar results for esophageal carcinoma (60), gastric cancer (61, 62), and rectal carcinoma (63). The present study provides further evidence that PNI is also associated with the outcomes of OTSCC. Of course, the magnitude of the association might vary among different types of cancer. Future studies could aim at quantifying these differences. In addition, the characteristics of PNI (i.e., size of the involved nerves, number of foci and involved nerves, and intratumoral or peritumoral localization) influence the prognostic significance of PNI (64–66). In the present meta-analysis, the characteristics of PNI were not consistently reported among the included studies, and the number of studies was too small for stratified analyses. Future studies should examine the characteristics of PNI more closely.

Hence, PNI is a prognostic marker in OTSCC. PNI could be used as a marker for more aggressive management in patients with OTSCC. Indeed, the presence of PNI has been suggested to guide the management of various cancers, like skin squamous and basal cell carcinoma (67), oral cancer (68), and colorectal cancer (69). Of note, Yang et al. (43) showed that elective neck dissection did not improve the prognosis in patients with OTSCC and PNI. This implies that surgery, which aims at macroscopic disease, might not be adequate for this type of OTSCC and that modalities targeting the microscopic disease, like radiotherapy and systemic therapy, might be more appropriate. Still, it will have to be examined in future studies.

This study has limitations. Firstly, even though 4445 patients were included in this study, the number of prospective studies was small, indicating that the results might be influenced by the biases inherited from the retrospective studies. In such instances, the quality of evidence of the analysis would be undermined. Secondly, we were interested in examining the impact of PNI on the survival outcomes among patients with OTSCC of different stages as we assumed that PNI could be incorporated as a reliable predictor into the current staging guidelines. Although a subgroup analysis for the stage was performed, there are a few points that need to be addressed before concluding about the assumption that PNI is associated with survival. First, only a few numbers of studies investigated early-stage OTSCC patients, resulting in low power. Besides, the guidelines used for defining cancer stages varied from the 6^th^ to the 8^th^ edition of the AJCC staging system, which might cause significant disparity among studies and affect the results. In addition, the publication bias could not be assessed because the number of studies included in each quantitative analysis was <10 (37, 42). Finally, the adjusted HRs were analyzed, but the covariates used for adjustment varied considerably among the included studies, probably contributing to heterogeneity and suggesting that the results should be taken with caution.

In conclusion, the presence of PNI significantly affects the locoregional recurrence and survival outcomes among patients with OTSCC. Extensive prospective studies should thoroughly investigate the impact of PNI in OTSCC as it is strongly affecting OTSCC locoregional recurrence and patient survival. The heterogeneity of the prognostic outcomes according to PNI among different cancer stages should be analyzed and discussed in the future.

## Data Availability Statement

The original contributions presented in the study are included in the article/**Supplementary Material**. Further inquiries can be directed to the corresponding author.

## Author Contributions

JL conceived and coordinated the study, designed, performed, and analyzed the experiments, wrote the paper. JL and SL carried out the data collection, data analysis and revised the paper. All authors contributed to the article and approved the submitted version.

## Funding

This study was supported by the Fundamental and Applied Fundamental Research Project of West China Hospital of Stomatology Sichuan University (Grant: RD-02-201909) and the National Natural Science Foundation of China (Grant: 81902775).

## Conflict of Interest

The authors declare that the research was conducted in the absence of any commercial or financial relationships that could be construed as a potential conflict of interest.

## Abbreviations

PNI, perineural invasion; OTSCC, oral tongue squamous cell carcinoma; HR, hazard ratio; OS, overall survival; DFS, disease-free survival; CSS, cancer-specific survival; OSCC, oral squamous cell carcinoma; HNSCC, head and neck squamous cell carcinoma; PRISMA, Preferred Reporting Items for Systematic Reviews and Meta-Analyses; CIs, confidence intervals; RCT, randomized controlled study; NOS, Newcastle-Ottawa Scale; MINORS, Methodological Items for Non-Randomized Studies.
