# Supplementary material for: Prognostic Value of Perineural Invasion in Oral Tongue Squamous Cell Carcinoma: A Systematic Review and Meta-Analysis
Source: Front Oncol. 2021 Jul 12;11:683825. doi: 10.3389/fonc.2021.683825 (PMC8311439; doi:10.3389/fonc.2021.683825)
Supplement: Supplementary file 1 [file DataSheet_1.docx]

**Supplementary Figures**


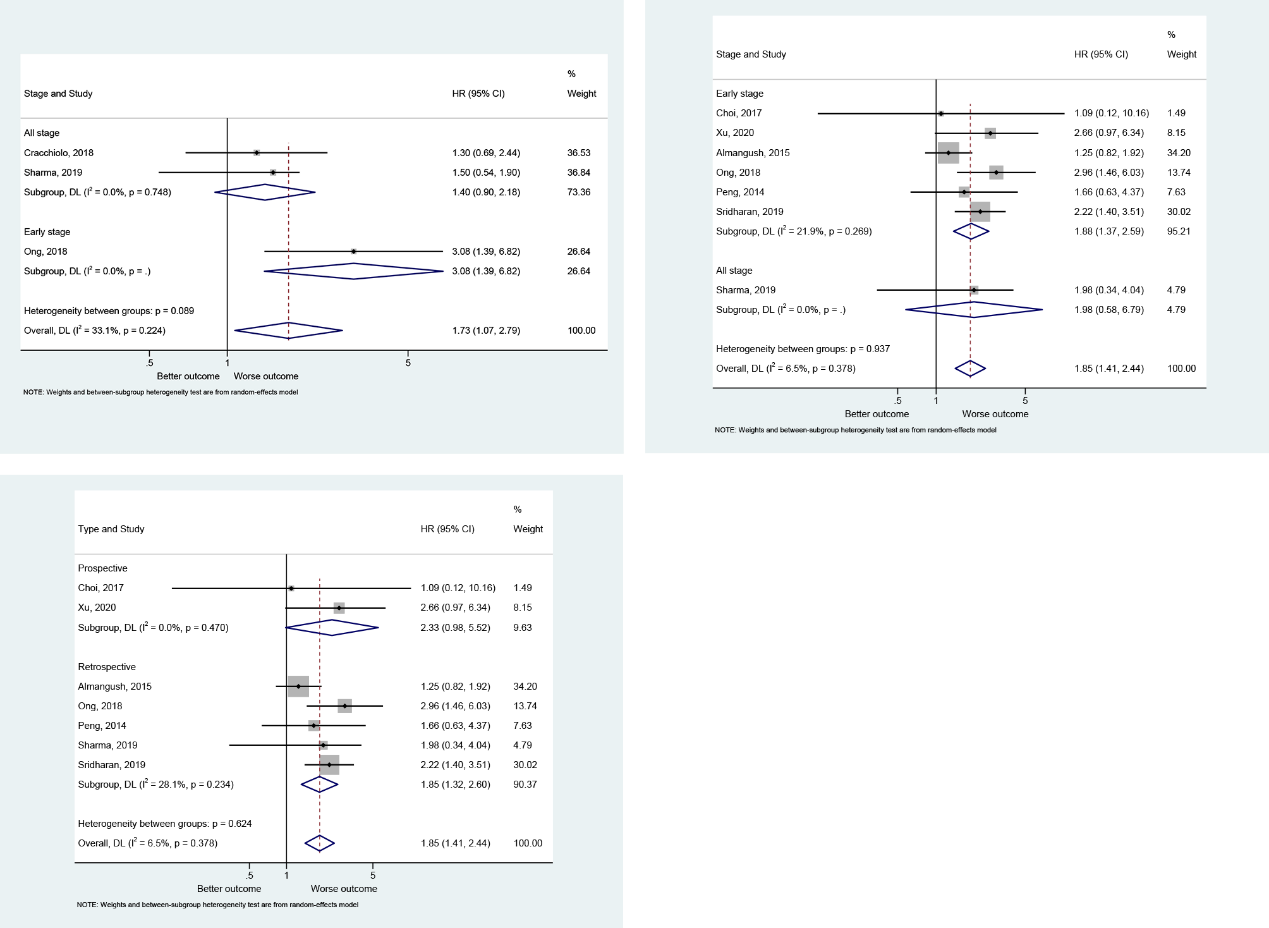


**Supplementary Figure S1.** Subgroup analysis of recurrence

**
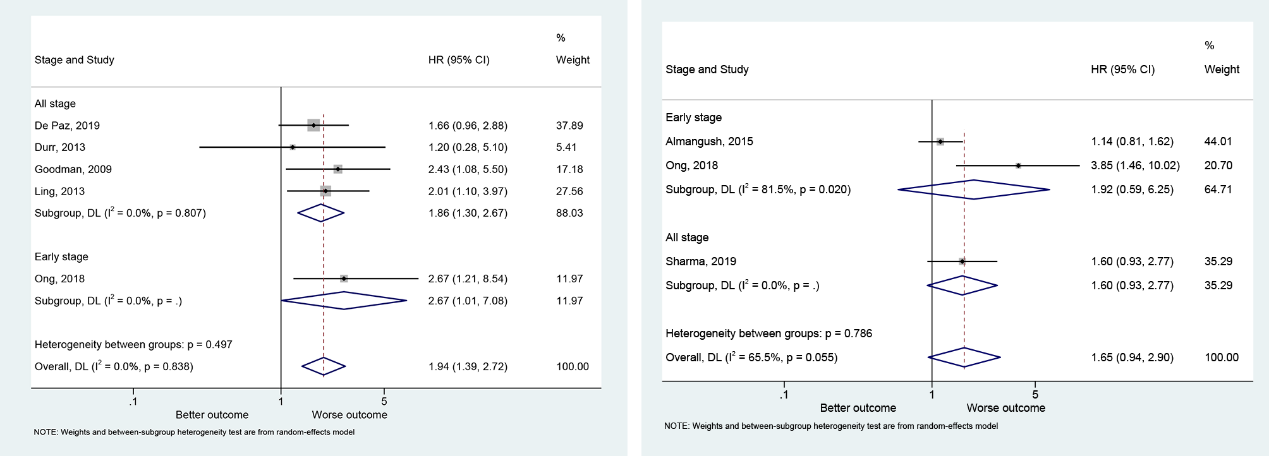
**

**Supplementary Figure S2.** Subgroup analysis of overall survival

**
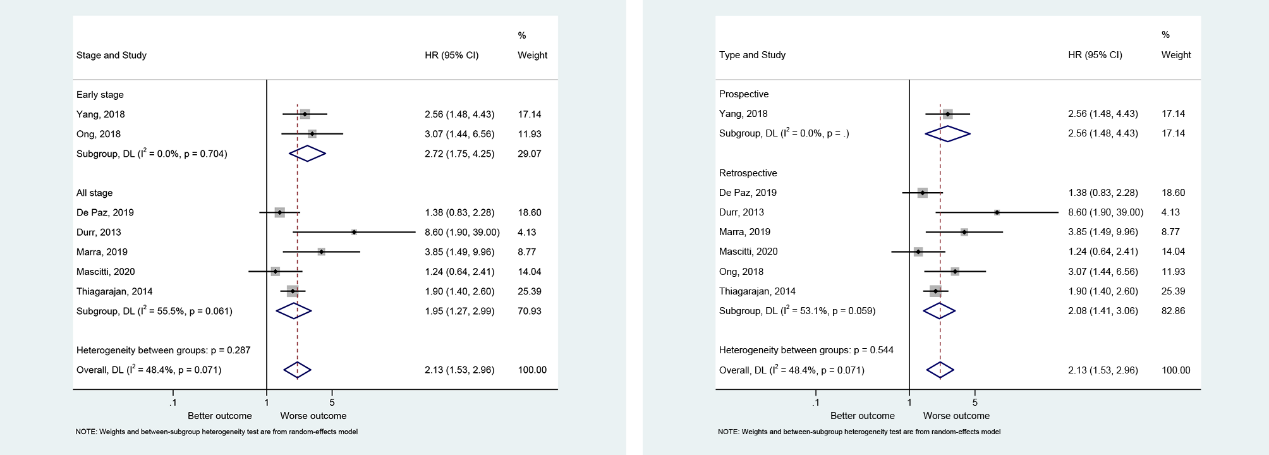
**

**Supplementary Figure S3.** Subgroup analysis of disease-free survival

**
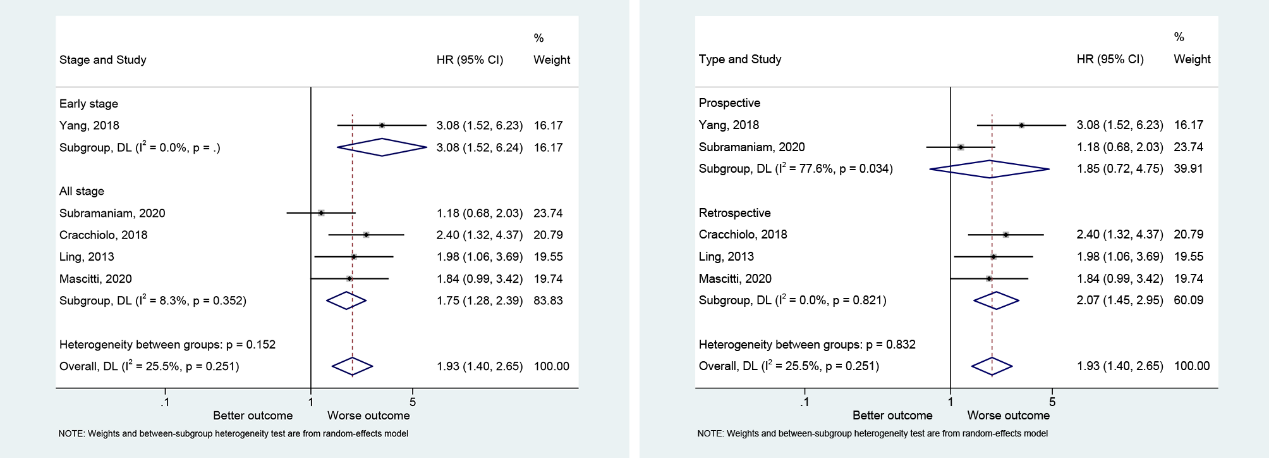
**

**Supplementary Figure S4.** Subgroup analysis of cancer-specific survival


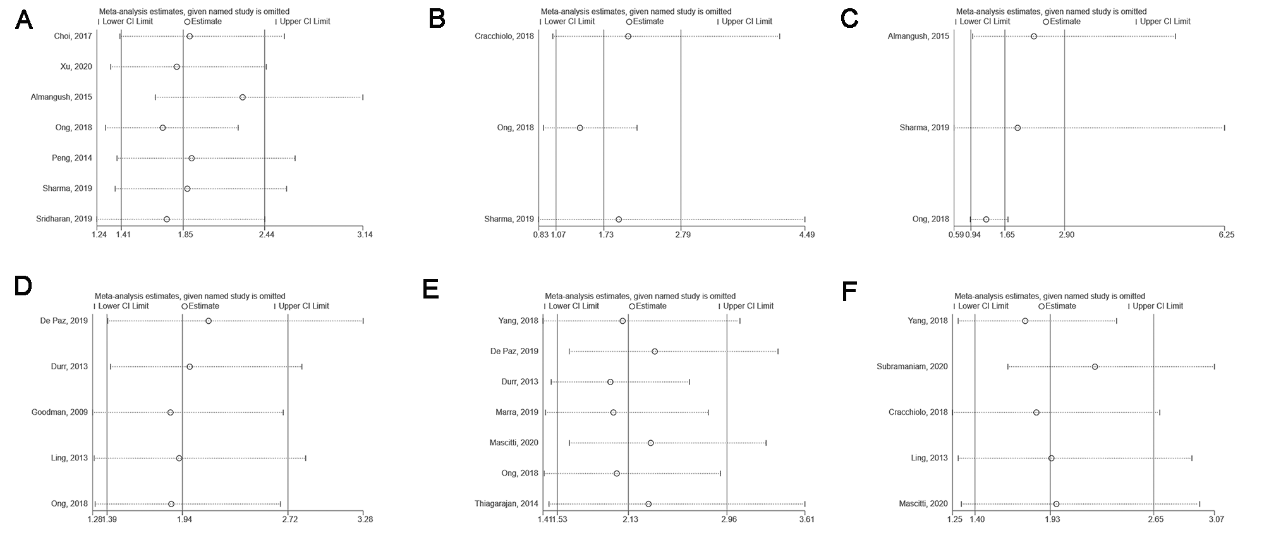


**Supplementary Figure S5.** Sensitivity analysis for (a) recurrence by unadjusted model, (b) recurrence by adjusted model (c) overall survival by unadjusted model, (d) overall survival by adjusted model, (e) disease-free survival, (f) cancer-specific survival.
