# Supplementary material for: Prognostic Value of Perineural Invasion in Oral Tongue Squamous Cell Carcinoma: A Systematic Review and Meta-Analysis
Source: Front Oncol. 2021 Jul 12;11:683825. doi: 10.3389/fonc.2021.683825 (PMC8311439; doi:10.3389/fonc.2021.683825)
Supplement: Supplementary file 2 [file Table_1.docx]

**Supplementary Table S1.** Search strategy

Date：2020/12/07

| **PubMed** |  | **Search strategy** | **Numbers** |
| --- | --- | --- | --- |
| **Patient** | **#1** | "Tongue Neoplasms"[Mesh] OR Neoplasm, Tongue OR Tongue Neoplasm OR Neoplasms, Tongue OR Cancer of Tongue OR Tongue Cancers OR Cancer of the Tongue OR Tongue Cancer OR Cancer, Tongue OR Cancers, Tongue | 17013 |
| **Intervention** | **#2** | perineural invasion OR perineural | 7869 |
| **Outcome** | **#3** | prognosis[MeSH:noexp] OR diagnosed[Title/Abstract] OR cohort*[Title/Abstract] OR cohort effect [MeSH] OR cohort studies [MeSH:noexp] OR predictor*[Title/Abstract] OR death[Title/Abstract] OR "models, statistical"[MeSH] OR Disease-free survival OR DFS OR OS OR Survival analysis | 3163064 |
| **All** | **#4** | #1 AND #2 AND #3 | 133 |

| **Embase** |  | **Search strategy** | **Numbers** |
| --- | --- | --- | --- |
| **Patient** | **#1** | 'tongue tumor'/exp OR 'lingual tumor' OR 'lingual tumour' OR 'tongue neoplasms' OR 'tongue tumour' OR 'tumor, lingual' OR 'tumor, tongue' OR 'tumour, lingual' OR 'tumour, tongue' | 13270 |
| **Intervention** | **#2** | 'perineural invasion'/exp OR 'perineural tumor invasion' OR 'perineural tumour invasion' | 5551 |
| **Outcome** | **#3** | 'prognosis'/exp OR 'prognosis' OR 'death'/exp OR 'death' OR 'disease free survival'/exp OR 'disease free survival' OR 'dfs (disease free survival)'/exp OR 'dfs (disease free survival)' OR 'disease-free survival'/exp OR 'disease-free survival' OR 'survival analysis'/exp OR 'survival analysis' OR 'os' | 2574602 |
| **All** | **#4** | #1 AND #2 AND #3 | 88 |

| **Cochrane** |  | **Search strategy** | **Numbers** |
| --- | --- | --- | --- |
| **Patient** | **#1** | MeSH descriptor: [Tongue Neoplasms] explode all trees | 64 |
|  | **#2** | (Cancers, Tongue) OR (Cancer, Tongue) OR (Cancer of the Tongue) OR (Cancer of Tongue) OR (Tongue Cancers) OR (Tongue Cancer) OR (Neoplasm, Tongue) OR (Tongue Neoplasm) OR (Neoplasms, Tongue) | 459 |
|  | **#3** | #1 OR #2 | 459 |
|  | **#4** | perineural invasion OR perineural | 849 |
|  | **#5** | #3 AND #4 | 6(5 trials) |

**Supplementary Table S2.** Reasons for exclusion of full text

| **Exclusion reasons** | **No.** |
| --- | --- |
| Study aim/design [1-13] | 13 |
| Population [14-64] | 51 |
| Outcomes [65-120] | 56 |
| Intervention [121-143] | 23 |
| Same population [144] with Cracchiolo, 2018 [145] | 1 |

1. Bisht N, Singh S, Mishra PS, Gupta S, Kapoor A. A rare case of endobronchial metastasis in a case of carcinoma tongue. Indian Journal of Medical and Paediatric Oncology. 2019;40(5):S181-S4. doi: 10.4103/ijmpo.ijmpo_205_17.

2. Saco M, Howe N, Jukic DM, Muro-Cacho C. Adenoid cystic carcinoma of the base of the tongue metastasizing to the scalp. Dermatology Online Journal. 2014;20(3).

3. Alabi RO, Elmusrati M, Sawazaki-Calone I, Kowalski LP, Haglund C, Coletta RD, et al. Machine learning application for prediction of locoregional recurrences in early oral tongue cancer: a Web-based prognostic tool. Virchows Archiv : an international journal of pathology. 2019;475(4):489-97. Epub 2019/08/20. doi: 10.1007/s00428-019-02642-5. PubMed PMID: 31422502; PubMed Central PMCID: PMCPMC6828835.

4. Bai L, Chang HM, Zhang L, Zhu YM, Leung PCK. BMP2 increases the production of BDNF through the upregulation of proBDNF and furin expression in human granulosa-lutein cells. FASEB J. 2020;34(12):16129-43. doi: 10.1096/fj.202000940R. PubMed PMID: 33047388.

5. González-García R, Naval-Gías L, Sastre-Pérez J, Rodríguez-Campo FJ, Muñoz-Guerra MF, Usandizaga JL, et al. Contralateral lymph neck node metastasis of primary squamous cell carcinoma of the tongue: a retrospective analytic study of 203 patients. International journal of oral and maxillofacial surgery. 2007;36(6):507-13. Epub 2007/03/03. doi: 10.1016/j.ijom.2007.01.008. PubMed PMID: 17331706.

6. Jani K, Balasubramanian D, Jayasankaran S, Murthy S, Vidyadaran S, Thankappan K, et al. Patterns of growth of lingual carcinoma on magnetic resonance imaging and correlations with clinicopathologic outcomes. Oral surgery, oral medicine, oral pathology and oral radiology. 2020;130(6):731-40. Epub 2020/07/23. doi: 10.1016/j.oooo.2020.06.012. PubMed PMID: 32693950.

7. Sinclair CF, Carroll WR, Desmond RA, Rosenthal EL. Functional and survival outcomes in patients undergoing total glossectomy compared with total laryngoglossectomy. Otolaryngology--head and neck surgery : official journal of American Academy of Otolaryngology-Head and Neck Surgery. 2011;145(5):755-8. Epub 2011/06/15. doi: 10.1177/0194599811412724. PubMed PMID: 21670476.

8. Subramaniam N, Balasubramanian D, Low TH, Murthy S, Clark JR, Thankappan K, et al. Factors Affecting Survival in Surgically Salvaged Locoregional Recurrences of Squamous Cell Carcinoma of the Tongue. Journal of oral and maxillofacial surgery : official journal of the American Association of Oral and Maxillofacial Surgeons. 2018;76(5):1133.e1-.e6. Epub 2018/02/07. doi: 10.1016/j.joms.2017.12.029. PubMed PMID: 29406254.

9. Yonezawa N, Minamikawa T, Kitajima K, Takahashi Y, Sasaki R, Nibu KI, et al. The maximum standardized uptake value increment calculated by dual-time-point (18)F-fluorodeoxyglucose positron emission tomography predicts survival in patients with oral tongue squamous cell carcinoma. Nagoya journal of medical science. 2017;79(2):189-98. Epub 2017/06/20. doi: 10.18999/nagjms.79.2.189. PubMed PMID: 28626254; PubMed Central PMCID: PMCPMC5472544.

10. Mobarki M, Dumollard JM, Dal Col P, Camy F, Peoc'h M, Karpathiou G. Granular cell tumor a study of 42 cases and systemic review of the literature. Pathology Research and Practice. 2020;216(4). doi: 10.1016/j.prp.2020.152865.

11. Ong HS, Gokavarapu S, Wang LZ, Tian Z, Zhang CP. Low Pretreatment Lymphocyte-Monocyte Ratio and High Platelet-Lymphocyte Ratio Indicate Poor Cancer Outcome in Early Tongue Cancer. Journal of Oral and Maxillofacial Surgery. 2017;75(8):1762-74. doi: 10.1016/j.joms.2016.12.023.

12. Shah D, Shah D, Kanhere S, Sanghavi K, Patel K. Role of imaging in clinically occult isolated intrathyroidal metastasis from squamous cell carcinoma of tongue: An unusual case series. Indian Journal of Nuclear Medicine. 2018;33(4):326-30. doi: 10.4103/ijnm.IJNM_106_18.

13. Subramaniam N, Balasubramanian D, Low THH, Murthy S, Clark JR, Thankappan K, et al. Factors Affecting Survival in Surgically Salvaged Locoregional Recurrences of Squamous Cell Carcinoma of the Tongue. Journal of Oral and Maxillofacial Surgery. 2018;76(5):1133.e1-.e6. doi: 10.1016/j.joms.2017.12.029.

14. Alvi A, Johnson JT. Development of distant metastasis after treatment of advanced-stage head and neck cancer. Head & neck. 1997;19(6):500-5. Epub 1997/09/01. doi: 10.1002/(sici)1097-0347(199709)19:6<500::aid-hed7>3.0.co;2-2. PubMed PMID: 9278758.

15. Anwer AW, Faisal M, Adeel M, Waqas O, Abu Bakar M, Qadeer S, et al. Clinicopathological Behavior and Treatment-related Outcome of Rare Salivary Duct Carcinoma: The Shaukat Khanum Memorial Cancer Hospital Experience. Cureus. 2018;10(8):e3139. Epub 2018/10/23. doi: 10.7759/cureus.3139. PubMed PMID: 30345196; PubMed Central PMCID: PMCPMC6188220.

16. Camisasca DR, Honorato J, Bernardo V, da Silva LE, da Fonseca EC, de Faria PA, et al. Expression of Bcl-2 family proteins and associated clinicopathologic factors predict survival outcome in patients with oral squamous cell carcinoma. Oral oncology. 2009;45(3):225-33. Epub 2008/08/22. doi: 10.1016/j.oraloncology.2008.05.021. PubMed PMID: 18715811.

17. Camisasca DR, Silami MA, Honorato J, Dias FL, de Faria PA, Lourenço Sde Q. Oral squamous cell carcinoma: clinicopathological features in patients with and without recurrence. ORL; journal for oto-rhino-laryngology and its related specialties. 2011;73(3):170-6. Epub 2011/06/01. doi: 10.1159/000328340. PubMed PMID: 21625192.

18. de Pontes Santos HB, de Morais EF, Moreira DGL, Marinho LCN, Galvão HC, de Almeida Freitas R. Neurofibromas of the oral and maxillofacial complex: A 48-year retrospective study. Journal of cutaneous pathology. 2020;47(3):202-6. Epub 2019/11/02. doi: 10.1111/cup.13605. PubMed PMID: 31675118.

19. Dik EA, Ipenburg NA, Kessler PA, van Es RJJ, Willems SM. The value of histological grading of biopsy and resection specimens in early stage oral squamous cell carcinomas. Journal of cranio-maxillo-facial surgery : official publication of the European Association for Cranio-Maxillo-Facial Surgery. 2018;46(6):1001-6. Epub 2018/05/02. doi: 10.1016/j.jcms.2018.03.019. PubMed PMID: 29709328.

20. Dik EA, Willems SM, Ipenburg NA, Adriaansens SO, Rosenberg AJ, van Es RJ. Resection of early oral squamous cell carcinoma with positive or close margins: relevance of adjuvant treatment in relation to local recurrence: margins of 3 mm as safe as 5 mm. Oral oncology. 2014;50(6):611-5. Epub 2014/03/19. doi: 10.1016/j.oraloncology.2014.02.014. PubMed PMID: 24630900.

21. Eliassen AM, Hauff SJ, Tang AL, Thomas DH, McHugh JB, Walline HM, et al. Head and neck squamous cell carcinoma in pregnant women. Head & neck. 2013;35(3):335-42. Epub 2012/03/17. doi: 10.1002/hed.22973. PubMed PMID: 22422571; PubMed Central PMCID: PMCPMC3399935.

22. Feng Z, Cheng A, Alzahrani S, Li B, Han Z, Ward BB. Elective Neck Dissection in T1N0M0 Oral Squamous Cell Carcinoma: When Is It Necessary? Journal of oral and maxillofacial surgery : official journal of the American Association of Oral and Maxillofacial Surgeons. 2020;78(12):2306-15. Epub 2020/07/31. doi: 10.1016/j.joms.2020.06.037. PubMed PMID: 32730759.

23. Garzino-Demo P, Zavattero E, Franco P, Fasolis M, Tanteri G, Mettus A, et al. Parameters and outcomes in 525 patients operated on for oral squamous cell carcinoma. Journal of cranio-maxillo-facial surgery : official publication of the European Association for Cranio-Maxillo-Facial Surgery. 2016;44(9):1414-21. Epub 2016/08/04. doi: 10.1016/j.jcms.2016.06.007. PubMed PMID: 27485718.

24. Ho YY, Wu TY, Cheng HC, Yang CC, Wu CH. The significance of tumor budding in oral cancer survival and its relevance to the eighth edition of the American Joint Committee on Cancer staging system. Head & neck. 2019;41(9):2991-3001. Epub 2019/04/24. doi: 10.1002/hed.25780. PubMed PMID: 31012518.

25. Jang JY, Kim MJ, Ryu G, Choi N, Ko YH, Jeong HS. Prediction of Lymph Node Metastasis by Tumor Dimension Versus Tumor Biological Properties in Head and Neck Squamous Cell Carcinomas. Cancer research and treatment : official journal of Korean Cancer Association. 2016;48(1):54-62. Epub 2015/03/13. doi: 10.4143/crt.2014.332. PubMed PMID: 25761475; PubMed Central PMCID: PMCPMC4720059.

26. Jardim JF, Francisco AL, Gondak R, Damascena A, Kowalski LP. Prognostic impact of perineural invasion and lymphovascular invasion in advanced stage oral squamous cell carcinoma. International journal of oral and maxillofacial surgery. 2015;44(1):23-8. Epub 2014/12/03. doi: 10.1016/j.ijom.2014.10.006. PubMed PMID: 25457832.

27. Kim RY, Helman JI, Braun TM, Ward BB. Increased Presence of Perineural Invasion in the Tongue and Floor of the Mouth: Could It Represent a More Aggressive Oral Squamous Cell Carcinoma, or Do Larger Aggressive Tumors Cause Perineural Invasion? Journal of oral and maxillofacial surgery : official journal of the American Association of Oral and Maxillofacial Surgeons. 2019;77(4):852-8. Epub 2018/08/25. doi: 10.1016/j.joms.2018.07.023. PubMed PMID: 30142323.

28. Lee DJ, Kwon MJ, Nam ES, Kwon JH, Kim JH, Rho YS, et al. Histopathologic predictors of lymph node metastasis and prognosis in tonsillar squamous cell carcinoma. Korean journal of pathology. 2013;47(3):203-10. Epub 2013/07/10. doi: 10.4132/KoreanJPathol.2013.47.3.203. PubMed PMID: 23837012; PubMed Central PMCID: PMCPMC3701815.

29. Leszczyńska M, Tokarski M, Jarmołowska-Jurczyszyn D, Kosikowski P, Szyfter W, Wierzbicka M. Adverse histopathological findings in glottic cancer with anterior commissure involvement. European archives of oto-rhino-laryngology : official journal of the European Federation of Oto-Rhino-Laryngological Societies (EUFOS) : affiliated with the German Society for Oto-Rhino-Laryngology - Head and Neck Surgery. 2015;272(8):1973-81. Epub 2015/03/22. doi: 10.1007/s00405-015-3594-9. PubMed PMID: 25794542.

30. Liao CT, Huang SF, Chen IH, Kang CJ, Lin CY, Fan KH, et al. Tongue and buccal mucosa carcinoma: is there a difference in outcome? Annals of surgical oncology. 2010;17(11):2984-91. Epub 2010/06/23. doi: 10.1245/s10434-010-1174-1. PubMed PMID: 20567919.

31. Liu SA, Wang CC, Jiang RS, Lee FY, Lin WJ, Lin JC. Pathological features and their prognostic impacts on oral cavity cancer patients among different subsites - A singe institute's experience in Taiwan. Scientific reports. 2017;7(1):7451. Epub 2017/08/09. doi: 10.1038/s41598-017-08022-w. PubMed PMID: 28785002; PubMed Central PMCID: PMCPMC5547072.

32. Liu Y, Li H, Qin L, Huang X, Su M, Han Z. Prognostic Factors in Malignant Sublingual Salivary Gland Tumors. Journal of oral and maxillofacial surgery : official journal of the American Association of Oral and Maxillofacial Surgeons. 2017;75(7):1542-8. Epub 2017/01/04. doi: 10.1016/j.joms.2016.12.010. PubMed PMID: 28041842.

33. Lohse I, Brothers SP. Pathogenesis and Treatment of Pancreatic Cancer Related Pain. Anticancer research. 2020;40(4):1789-96. Epub 2020/04/03. doi: 10.21873/anticanres.14133. PubMed PMID: 32234867; PubMed Central PMCID: PMCPMC7323503.

34. Luna-Ortiz K, Carmona-Luna T, Cano-Valdez AM, Mosqueda-Taylor A, Herrera-Gómez A, Villavicencio-Valencia V. Adenoid cystic carcinoma of the tongue--clinicopathological study and survival analysis. Head & neck oncology. 2009;1:15. Epub 2009/06/02. doi: 10.1186/1758-3284-1-15. PubMed PMID: 19480697; PubMed Central PMCID: PMCPMC2694803.

35. Magnano M, Bongioannini G, Lerda W, Canale G, Tondolo E, Bona M, et al. Lymphnode metastasis in head and neck squamous cells carcinoma: multivariate analysis of prognostic variables. Journal of experimental & clinical cancer research : CR. 1999;18(1):79-83. Epub 1999/06/22. PubMed PMID: 10374683.

36. Magnano M, De Stefani A, Lerda W, Usai A, Ragona R, Bussi M, et al. Prognostic factors of cervical lymph node metastasis in head and neck squamous cell carcinoma. Tumori. 1997;83(6):922-6. Epub 1998/04/04. PubMed PMID: 9526585.

37. Maher NG, Hoffman GR. Elective neck dissection for primary oral cavity squamous cell carcinoma involving the tongue should include sublevel IIb. Journal of oral and maxillofacial surgery : official journal of the American Association of Oral and Maxillofacial Surgeons. 2014;72(11):2333-43. Epub 2014/08/31. doi: 10.1016/j.joms.2014.05.022. PubMed PMID: 25172672.

38. Monteiro LS, Amaral JB, Vizcaíno JR, Lopes CA, Torres FO. A clinical-pathological and survival study of oral squamous cell carcinomas from a population of the North of Portugal. Medicina oral, patologia oral y cirugia bucal. 2014;19(2):e120-6. Epub 2013/10/15. doi: 10.4317/medoral.19090. PubMed PMID: 24121907; PubMed Central PMCID: PMCPMC4015041.

39. Nair D, Mair M, Singhvi H, Mishra A, Nair S, Agrawal J, et al. Perineural invasion: Independent prognostic factor in oral cancer that warrants adjuvant treatment. Head & neck. 2018;40(8):1780-7. Epub 2018/05/01. doi: 10.1002/hed.25170. PubMed PMID: 29707840.

40. Nemec A, Murphy B, Kass PH, Verstraete FJ. Histological subtypes of oral non-tonsillar squamous cell carcinoma in dogs. Journal of comparative pathology. 2012;147(2-3):111-20. Epub 2012/02/04. doi: 10.1016/j.jcpa.2011.11.198. PubMed PMID: 22300705.

41. Pandey M, Kannepali KK, Dixit R, Kumar M. Effect of neoadjuvant chemotherapy and its correlation with HPV status, EGFR, Her-2-neu, and GADD45 expression in oral squamous cell carcinoma. World journal of surgical oncology. 2018;16(1):20. Epub 2018/02/02. doi: 10.1186/s12957-018-1308-7. PubMed PMID: 29386013; PubMed Central PMCID: PMCPMC5793383.

42. Rodrigues RM, Bernardo VG, Da Silva SD, Camisasca DR, Faria PAS, Dias FL, et al. How pathological criteria can impact prognosis of tongue and floor of the mouth squamous cell carcinoma. Journal of applied oral science : revista FOB. 2020;28:e20190198. Epub 2019/12/05. doi: 10.1590/1678-7757-2019-0198. PubMed PMID: 31800876; PubMed Central PMCID: PMCPMC6886392.

43. Rubio Bueno P, Naval Gias L, García Delgado R, Domingo Cebollada J, Díaz González FJ. Tumor DNA content as a prognostic indicator in squamous cell carcinoma of the oral cavity and tongue base. Head & neck. 1998;20(3):232-9. Epub 1998/05/07. doi: 10.1002/(sici)1097-0347(199805)20:3<232::aid-hed8>3.0.co;2-1. PubMed PMID: 9570629.

44. Sim YC, Hwang JH, Ahn KM. Overall and disease-specific survival outcomes following primary surgery for oral squamous cell carcinoma: analysis of consecutive 67 patients. Journal of the Korean Association of Oral and Maxillofacial Surgeons. 2019;45(2):83-90. Epub 2019/05/21. doi: 10.5125/jkaoms.2019.45.2.83. PubMed PMID: 31106136; PubMed Central PMCID: PMCPMC6502750.

45. Sinha N, Rigby MH, McNeil ML, Taylor SM, Trites JR, Hart RD, et al. The histologic risk model is a useful and inexpensive tool to assess risk of recurrence and death in stage I or II squamous cell carcinoma of tongue and floor of mouth. Modern pathology : an official journal of the United States and Canadian Academy of Pathology, Inc. 2018;31(5):772-9. Epub 2018/02/03. doi: 10.1038/modpathol.2017.183. PubMed PMID: 29393297.

46. Sinha P, Hackman T, Nussenbaum B, Wu N, Lewis JS, Jr., Haughey BH. Transoral laser microsurgery for oral squamous cell carcinoma: oncologic outcomes and prognostic factors. Head & neck. 2014;36(3):340-51. Epub 2013/06/05. doi: 10.1002/hed.23293. PubMed PMID: 23729304; PubMed Central PMCID: PMCPMC3951113.

47. Subramaniam N, Balasubramanian D, Murthy S, Kumar N, Vidhyadharan S, Vijayan SN, et al. Predictors of locoregional control in stage I/II oral squamous cell carcinoma classified by AJCC 8th edition. European journal of surgical oncology : the journal of the European Society of Surgical Oncology and the British Association of Surgical Oncology. 2019;45(11):2126-30. Epub 2019/06/15. doi: 10.1016/j.ejso.2019.05.018. PubMed PMID: 31196702.

48. Tai SK, Li WY, Yang MH, Chu PY, Wang YF, Chang PM. Perineural invasion as a major determinant for the aggressiveness associated with increased tumor thickness in t1-2 oral tongue and buccal squamous cell carcinoma. Ann Surg Oncol. 2013;20(11):3568-74. Epub 2013/07/11. doi: 10.1245/s10434-013-3068-5. PubMed PMID: 23838906.

49. van Es RJ, van Nieuw Amerongen N, Slootweg PJ, Egyedi P. Resection margin as a predictor of recurrence at the primary site for T1 and T2 oral cancers. Evaluation of histopathologic variables. Archives of otolaryngology--head & neck surgery. 1996;122(5):521-5. Epub 1996/05/01. doi: 10.1001/archotol.1996.01890170055011. PubMed PMID: 8615970.

50. Weijers M, Snow GB, Bezemer DP, van dr Wal JE, van der Waal I. The status of the deep surgical margins in tongue and floor of mouth squamous cell carcinoma and risk of local recurrence; an analysis of 68 patients. International journal of oral and maxillofacial surgery. 2004;33(2):146-9. Epub 2004/03/31. doi: 10.1054/ijom.2002.0469. PubMed PMID: 15050070.

51. Woolgar JA, Scott J. Prediction of cervical lymph node metastasis in squamous cell carcinoma of the tongue/floor of mouth. Head & neck. 1995;17(6):463-72. Epub 1995/11/01. doi: 10.1002/hed.2880170603. PubMed PMID: 8847204.

52. Adel M, Liao CT, Lee LY, Hsueh C, Lin CY, Fan KH, et al. Incidence and outcomes of patients with oral cavity squamous cell Carcinoma and fourth primary tumors a long-term follow-up study in a betel quid chewing endemic area. Medicine (United States). 2016;95(12). doi: 10.1097/MD.0000000000002950.

53. Cao Y, Li R, Cheng L, Chen N, Li J, Yu D. P75 Nerve Growth Factor Receptor as a Specific Nerve Marker in the Diagnosis of Perineural Invasion of Squamous Cell Carcinoma. American Journal of Clinical Pathology. 2019;151(6):574-83. doi: 10.1093/ajcp/aqz011.

54. Chen YW, Chen IL, Lin IC, Kao SY. Prognostic value of hypercalcaemia and leucocytosis in resected oral squamous cell carcinoma. British Journal of Oral and Maxillofacial Surgery. 2014;52(5):425-31. doi: 10.1016/j.bjoms.2014.02.014.

55. Cho Y, Yoon HI, Lee IJ, Kim JW, Lee CG, Choi EC, et al. Patterns of local recurrence after curative resection and reconstruction for oropharyngeal and oral cancers: Implications for postoperative radiotherapy target volumes. Head and Neck. 2019;41(11):3916-23. doi: 10.1002/hed.25928.

56. Domingueti CB, Janini JBM, Paranaíba LMR, Lozano-Burgos C, Olivero P, González-Arriagada WA. Prognostic value of immunoexpression of ccr4, ccr5, ccr7 and cxcr4 in squamous cell carcinoma of tongue and floor of the mouth. Medicina Oral Patologia Oral y Cirugia Bucal. 2019;24(3):e354-e63. doi: 10.4317/medoral.22904.

57. Iqbal MS, Paleri V, Brown J, Greystoke A, Dobrowsky W, Kelly C, et al. Spindle cell carcinoma of the head and neck region: Treatment and outcomes of 15 patients. ecancermedicalscience. 2015;9. doi: 10.3332/ecancer.2015.594.

58. López-Cedrún JL, Andrés De Llano J. A 22 years survival and prognostic factors analysis in a homogeneous series of 64 patients with advanced cancer of the tongue and the floor of the mouth. Journal of Cranio-Maxillofacial Surgery. 2015;43(3):376-81. doi: 10.1016/j.jcms.2015.01.007.

59. Moratin J, Metzger K, Kansy K, Ristow O, Engel M, Hoffmann J, et al. The prognostic significance of the lymph node ratio in oral cancer differs for anatomical subsites. International journal of oral and maxillofacial surgery. 2020;49(5):558-63. doi: 10.1016/j.ijom.2019.10.015.

60. Park S, Nam SJ, Keam B, Kim TM, Jeon YK, Lee SH, et al. VEGF and Ki-67 overexpression in predicting poor overall survival in adenoid cystic carcinoma. Cancer Research and Treatment. 2016;48(2):518-26. doi: 10.4143/crt.2015.093.

61. Pedersen NJ, Jensen DH, Hedbäck N, Frendø M, Kiss K, Lelkaitis G, et al. Staging of early lymph node metastases with the sentinel lymph node technique and predictive factors in T1/T2 oral cavity cancer: A retrospective single-center study. Head and Neck. 2016;38:E1033-E40. doi: 10.1002/hed.24153.

62. Roy S, Kar M, Roy S, Saha A, Padhi S, Banerjee B. Role of β-catenin in cisplatin resistance, relapse and prognosis of head and neck squamous cell carcinoma. Cellular Oncology. 2018;41(2):185-200. doi: 10.1007/s13402-017-0365-1.

63. Samant S. Sentinel node biopsy as an alternative to elective neck dissection for staging of early oral carcinoma. Head and Neck. 2014;36(2):241-6. doi: 10.1002/hed.23288.

64. Yamagata K, Fukuzawa S, Kanno N, Uchida F, Yanagawa T, Bukawa H. Is Lymph Node Ratio a Prognostic Factor for Patients With Oral Squamous Cell Carcinoma? Journal of Oral and Maxillofacial Surgery. 2019;77(7):1510-9. doi: 10.1016/j.joms.2019.01.037.

65. Almangush A, Mäkitie AA, Hagström J, Haglund C, Kowalski LP, Nieminen P, et al. Cell-in-cell phenomenon associates with aggressive characteristics and cancer-related mortality in early oral tongue cancer. BMC cancer. 2020;20(1):843. Epub 2020/09/05. doi: 10.1186/s12885-020-07342-x. PubMed PMID: 32883229; PubMed Central PMCID: PMCPMC7469910.

66. Asakage T, Yokose T, Mukai K, Tsugane S, Tsubono Y, Asai M, et al. Tumor thickness predicts cervical metastasis in patients with stage I/II carcinoma of the tongue. Cancer. 1998;82(8):1443-8. Epub 1998/04/29. doi: 10.1002/(sici)1097-0142(19980415)82:8<1443::aid-cncr2>3.0.co;2-a. PubMed PMID: 9554518.

67. Borges AM, Shrikhande SS, Ganesh B. Surgical pathology of squamous carcinoma of the oral cavity: its impact on management. Seminars in surgical oncology. 1989;5(5):310-7. Epub 1989/01/01. doi: 10.1002/ssu.2980050504. PubMed PMID: 2814140.

68. Brandwein-Gensler M, Teixeira MS, Lewis CM, Lee B, Rolnitzky L, Hille JJ, et al. Oral squamous cell carcinoma: histologic risk assessment, but not margin status, is strongly predictive of local disease-free and overall survival. The American journal of surgical pathology. 2005;29(2):167-78. Epub 2005/01/13. doi: 10.1097/01.pas.0000149687.90710.21. PubMed PMID: 15644773.

69. Brennan S, Corry J, Kleid S, Porceddu S, Yuen K, Rischin D, et al. Prospective trial to evaluate staged neck dissection or elective neck radiotherapy in patients with CT-staged T1-2 N0 squamous cell carcinoma of the oral tongue. Head & neck. 2010;32(2):191-8. Epub 2009/07/03. doi: 10.1002/hed.21167. PubMed PMID: 19572285.

70. Cassidy RJ, Switchenko JM, Jegadeesh N, Sayan M, Ferris MJ, Eaton BR, et al. Association of Lymphovascular Space Invasion With Locoregional Failure and Survival in Patients With Node-Negative Oral Tongue Cancers. JAMA otolaryngology-- head & neck surgery. 2017;143(4):382-8. doi: 10.1001/jamaoto.2016.3795. PubMed PMID: 28097311; PubMed Central PMCID: PMCPMC5398912.

71. Chatterjee D, Bansal V, Malik V, Bhagat R, Punia RS, Handa U, et al. Tumor Budding and Worse Pattern of Invasion Can Predict Nodal Metastasis in Oral Cancers and Associated With Poor Survival in Early-Stage Tumors. Ear, nose, & throat journal. 2019;98(7):E112-e9. Epub 2019/05/11. doi: 10.1177/0145561319848669. PubMed PMID: 31072197.

72. Chatzistamou I, Rodriguez J, Jouffroy T, Girod A, Point D, Sklavounou A, et al. Prognostic significance of tumor shape and stromal chronic inflammatory infiltration in squamous cell carcinomas of the oral tongue. Journal of oral pathology & medicine : official publication of the International Association of Oral Pathologists and the American Academy of Oral Pathology. 2010;39(9):667-71. Epub 2010/07/14. doi: 10.1111/j.1600-0714.2010.00911.x. PubMed PMID: 20618607.

73. Chen YW, Yu EH, Wu TH, Lo WL, Li WY, Kao SY. Histopathological factors affecting nodal metastasis in tongue cancer: analysis of 94 patients in Taiwan. International journal of oral and maxillofacial surgery. 2008;37(10):912-6. Epub 2008/09/16. doi: 10.1016/j.ijom.2008.07.014. PubMed PMID: 18789650.

74. Chuang HC, Su CY, Huang HY, Chien CY, Chen CM, Huang CC. High expression of CD105 as a prognostic predictor of early tongue cancer. The Laryngoscope. 2006;116(7):1175-9. Epub 2006/07/11. doi: 10.1097/01.mlg.0000224338.56902.28. PubMed PMID: 16826056.

75. Chung MK, Min JY, So YK, Ko YH, Jeong HS, Son YI, et al. Correlation between lymphatic vessel density and regional metastasis in squamous cell carcinoma of the tongue. Head & neck. 2010;32(4):445-51. Epub 2009/08/13. doi: 10.1002/hed.21202. PubMed PMID: 19672869.

76. de Matos FR, Lima E, Queiroz LM, da Silveira EJ. Analysis of inflammatory infiltrate, perineural invasion, and risk score can indicate concurrent metastasis in squamous cell carcinoma of the tongue. Journal of oral and maxillofacial surgery : official journal of the American Association of Oral and Maxillofacial Surgeons. 2012;70(7):1703-10. Epub 2011/12/14. doi: 10.1016/j.joms.2011.08.023. PubMed PMID: 22154400.

77. Doumas S, Paterson JC, Norris PM, Tighe JV, Newman L, Bisase BS, et al. Fractalkine (CX3CL1) and fractalkine receptor (CX3CR1) in squamous cell carcinoma of the tongue: markers of nerve invasion? Oral and maxillofacial surgery. 2015;19(1):61-4. Epub 2014/07/11. doi: 10.1007/s10006-014-0455-4. PubMed PMID: 25008036.

78. Fukano H, Matsuura H, Hasegawa Y, Nakamura S. Depth of invasion as a predictive factor for cervical lymph node metastasis in tongue carcinoma. Head & neck. 1997;19(3):205-10. Epub 1997/05/01. doi: 10.1002/(sici)1097-0347(199705)19:3<205::aid-hed7>3.0.co;2-6. PubMed PMID: 9142520.

79. Han N, Ong H, Liu Z, Ruan M, Yang W, Zhang C. Lymph node involvement predicts poor prognosis in primary tongue adenoid cystic carcinoma: A preliminary study of 54 cases. Journal of cranio-maxillo-facial surgery : official publication of the European Association for Cranio-Maxillo-Facial Surgery. 2017;45(4):589-94. Epub 2017/02/23. doi: 10.1016/j.jcms.2017.01.012. PubMed PMID: 28223016.

80. Hechler B, Carlson ER, Heidel RE, Fahmy MD, McCoy JM. Are Oral Pain and Otalgia Predictive of Perineural Invasion in Squamous Cell Carcinoma of the Oral Tongue? Journal of oral and maxillofacial surgery : official journal of the American Association of Oral and Maxillofacial Surgeons. 2020;78(8):1418-26. Epub 2020/05/04. doi: 10.1016/j.joms.2020.03.029. PubMed PMID: 32360237.

81. Hilly O, Shkedy Y, Hod R, Soudry E, Mizrachi A, Hamzany Y, et al. Carcinoma of the oral tongue in patients younger than 30 years: comparison with patients older than 60 years. Oral oncology. 2013;49(10):987-90. Epub 2013/08/10. doi: 10.1016/j.oraloncology.2013.07.005. PubMed PMID: 23927849.

82. Hingsammer L, Seier T, Ikenberg J, Schumann P, Zweifel D, Rücker M, et al. The influence of lymph node ratio on survival and disease recurrence in squamous cell carcinoma of the tongue. International journal of oral and maxillofacial surgery. 2019;48(7):851-6. Epub 2019/02/11. doi: 10.1016/j.ijom.2019.01.008. PubMed PMID: 30738712.

83. Iseli TA, Lin MJ, Tsui A, Guiney A, Wiesenfeld D, Iseli CE. Are wider surgical margins needed for early oral tongue cancer? The Journal of laryngology and otology. 2012;126(3):289-94. Epub 2012/01/20. doi: 10.1017/s002221511100332x. PubMed PMID: 22258616.

84. Jacob TE, Malathi N, Rajan ST, Augustine D, Manish N, Patil S. Histopathological Parameters predicting Occult Nodal Metastases in Tongue Carcinoma Cases: An Indian Perspective. The journal of contemporary dental practice. 2016;17(1):70-7. Epub 2016/04/17. doi: 10.5005/jp-journals-10024-1805. PubMed PMID: 27084866.

85. Katna R, Bhosale B, Sharma R, Singh S, Deshpande A, Kalyani N. Oncological outcomes in patients undergoing major glossectomy for advanced carcinoma of the oral tongue. Annals of the Royal College of Surgeons of England. 2020;102(7):514-8. Epub 2020/05/22. doi: 10.1308/rcsann.2020.0100. PubMed PMID: 32436723; PubMed Central PMCID: PMCPMC7450443.

86. Katz O, Nachalon Y, Hilly O, Shpitzer T, Bachar G, Limon D, et al. Radiotherapy in early-stage tongue squamous cell carcinoma with minor adverse features. Head & neck. 2017;39(1):147-50. Epub 2016/08/11. doi: 10.1002/hed.24555. PubMed PMID: 27507221.

87. Kolokythas A, Cox DP, Dekker N, Schmidt BL. Nerve growth factor and tyrosine kinase A receptor in oral squamous cell carcinoma: is there an association with perineural invasion? Journal of oral and maxillofacial surgery : official journal of the American Association of Oral and Maxillofacial Surgeons. 2010;68(6):1290-5. Epub 2010/04/07. doi: 10.1016/j.joms.2010.01.006. PubMed PMID: 20363547.

88. Limongelli L, Capodiferro S, Tempesta A, Sportelli P, Dell'Olio F, Angelelli G, et al. Early tongue carcinomas (clinical stage I and II): echo-guided three-dimensional diode laser mini-invasive surgery with evaluation of histological prognostic parameters. A study of 85 cases with prolonged follow-up. Lasers in medical science. 2020;35(3):751-8. Epub 2019/12/14. doi: 10.1007/s10103-019-02932-z. PubMed PMID: 31834561.

89. Lydiatt DD, Robbins KT, Byers RM, Wolf PF. Treatment of stage I and II oral tongue cancer. Head & neck. 1993;15(4):308-12. Epub 1993/07/01. doi: 10.1002/hed.2880150407. PubMed PMID: 8360052.

90. Maddox WA. Hayes Martin lecture. Vicissitudes of head and neck cancer. American journal of surgery. 1984;148(4):428-32. Epub 1984/10/01. doi: 10.1016/0002-9610(84)90363-5. PubMed PMID: 6486306.

91. Maddox WA, Urist MM. Histopathological prognostic factors of certain primary oral cavity cancers. Oncology (Williston Park, NY). 1990;4(12):39-42; discussion , 5-6. Epub 1990/12/01. PubMed PMID: 2149038.

92. Matsushita Y, Yanamoto S, Takahashi H, Yamada S, Naruse T, Sakamoto Y, et al. A clinicopathological study of perineural invasion and vascular invasion in oral tongue squamous cell carcinoma. International journal of oral and maxillofacial surgery. 2015;44(5):543-8. Epub 2015/02/24. doi: 10.1016/j.ijom.2015.01.018. PubMed PMID: 25697063.

93. Maxwell JH, Thompson LD, Brandwein-Gensler MS, Weiss BG, Canis M, Purgina B, et al. Early Oral Tongue Squamous Cell Carcinoma: Sampling of Margins From Tumor Bed and Worse Local Control. JAMA otolaryngology-- head & neck surgery. 2015;141(12):1104-10. Epub 2015/08/01. doi: 10.1001/jamaoto.2015.1351. PubMed PMID: 26225798; PubMed Central PMCID: PMCPMC5242089.

94. Miller C, Shay A, Tajudeen B, Sen N, Fidler M, Stenson K, et al. Clinical features and outcomes in young adults with oral tongue cancer. American journal of otolaryngology. 2019;40(1):93-6. Epub 2018/11/26. doi: 10.1016/j.amjoto.2018.09.022. PubMed PMID: 30472130.

95. Morton RP, Ferguson CM, Lambie NK, Whitlock RM. Tumor thickness in early tongue cancer. Archives of otolaryngology--head & neck surgery. 1994;120(7):717-20. Epub 1994/07/01. doi: 10.1001/archotol.1994.01880310023005. PubMed PMID: 8018323.

96. Myers JN, Elkins T, Roberts D, Byers RM. Squamous cell carcinoma of the tongue in young adults: increasing incidence and factors that predict treatment outcomes. Otolaryngology--head and neck surgery : official journal of American Academy of Otolaryngology-Head and Neck Surgery. 2000;122(1):44-51. Epub 2000/01/12. doi: 10.1016/s0194-5998(00)70142-2. PubMed PMID: 10629481.

97. O'Brien CJ, Lahr CJ, Soong SJ, Gandour MJ, Jones JM, Urist MM, et al. Surgical treatment of early-stage carcinoma of the oral tongue--wound adjuvant treatment be beneficial? Head & neck surgery. 1986;8(6):401-8. Epub 1986/07/01. doi: 10.1002/hed.2890080603. PubMed PMID: 3721882.

98. Perez-Ordonez B, Linkov I, Huvos AG. Polymorphous low-grade adenocarcinoma of minor salivary glands: a study of 17 cases with emphasis on cell differentiation. Histopathology. 1998;32(6):521-9. Epub 1998/07/24. doi: 10.1046/j.1365-2559.1998.t01-2-00410.x. PubMed PMID: 9675591.

99. Ryu HJ, Kim EK, Cho BC, Yoon SO. Characterization of head and neck squamous cell carcinoma arising in young patients: Particular focus on molecular alteration and tumor immunity. Head & neck. 2019;41(1):198-207. Epub 2018/12/12. doi: 10.1002/hed.25507. PubMed PMID: 30536665.

100. Safi AF, Grandoch A, Nickenig HJ, Zöller JE, Kreppel M. The importance of lymph node ratio for locoregional recurrence of squamous cell carcinoma of the tongue. Journal of cranio-maxillo-facial surgery : official publication of the European Association for Cranio-Maxillo-Facial Surgery. 2017;45(7):1058-61. Epub 2017/05/23. doi: 10.1016/j.jcms.2017.04.008. PubMed PMID: 28529103.

101. Singh A, Singhavi H, Sathe P, Mair M, Qayyumi B, Shetty R, et al. The impact of peritumoral depapillation in cancers of the tongue. Oral surgery, oral medicine, oral pathology and oral radiology. 2020;129(4):369-76. Epub 2020/02/10. doi: 10.1016/j.oooo.2019.12.016. PubMed PMID: 32035858.

102. Soudry E, Preis M, Hod R, Hamzany Y, Hadar T, Bahar G, et al. Squamous cell carcinoma of the oral tongue in patients younger than 30 years: clinicopathologic features and outcome. Clinical otolaryngology : official journal of ENT-UK ; official journal of Netherlands Society for Oto-Rhino-Laryngology & Cervico-Facial Surgery. 2010;35(4):307-12. Epub 2010/08/27. doi: 10.1111/j.1749-4486.2010.02164.x. PubMed PMID: 20738340.

103. Sparano A, Weinstein G, Chalian A, Yodul M, Weber R. Multivariate predictors of occult neck metastasis in early oral tongue cancer. Otolaryngology--head and neck surgery : official journal of American Academy of Otolaryngology-Head and Neck Surgery. 2004;131(4):472-6. Epub 2004/10/07. doi: 10.1016/j.otohns.2004.04.008. PubMed PMID: 15467620.

104. Subramaniam N, Balasubramanian D, Low TH, Murthy S, Anand A, Prasad C, et al. Role of adverse pathological features in surgically treated early oral cavity carcinomas with adequate margins and the development of a scoring system to predict local control. Head & neck. 2018;40(11):2329-33. Epub 2018/11/02. doi: 10.1002/hed.25163. PubMed PMID: 30381858.

105. Tai SK, Li WY, Chu PY, Chang SY, Tsai TL, Wang YF, et al. Risks and clinical implications of perineural invasion in T1-2 oral tongue squamous cell carcinoma. Head & neck. 2012;34(7):994-1001. Epub 2011/09/29. doi: 10.1002/hed.21846. PubMed PMID: 21953773.

106. Unal OF, Ayhan A, Hoşal AS. Prognostic value of p53 expression and histopathological parameters in squamous cell carcinoma of oral tongue. The Journal of laryngology and otology. 1999;113(5):446-50. Epub 1999/10/03. doi: 10.1017/s0022215100144184. PubMed PMID: 10505159.

107. Vered M, Dayan D, Dobriyan A, Yahalom R, Shalmon B, Barshack I, et al. Oral tongue squamous cell carcinoma: recurrent disease is associated with histopathologic risk score and young age. Journal of cancer research and clinical oncology. 2010;136(7):1039-48. Epub 2010/01/08. doi: 10.1007/s00432-009-0749-3. PubMed PMID: 20054559.

108. Vered M, Dobriyan A, Dayan D, Yahalom R, Talmi YP, Bedrin L, et al. Tumor-host histopathologic variables, stromal myofibroblasts and risk score, are significantly associated with recurrent disease in tongue cancer. Cancer science. 2010;101(1):274-80. Epub 2009/10/07. doi: 10.1111/j.1349-7006.2009.01357.x. PubMed PMID: 19804423.

109. Almangush A, Bello IO, Keski-Säntti H, Mäkinen LK, Kauppila JH, Pukkila M, et al. Depth of invasion, tumor budding, and worst pattern of invasion: Prognostic indicators in early-stage oral tongue cancer. Head and Neck. 2014;36(6):811-8. doi: 10.1002/hed.23380.

110. Almangush A, Coletta RD, Bello IO, Bitu C, Keski-Säntti H, Mäkinen LK, et al. A simple novel prognostic model for early stage oral tongue cancer. International journal of oral and maxillofacial surgery. 2015;44(2):143-50. doi: 10.1016/j.ijom.2014.10.004.

111. Carta F, Quartu D, Mariani C, Tatti M, Marrosu V, Gioia E, et al. Compartmental Surgery With Microvascular Free Flap Reconstruction in Patients With T1–T4 Squamous Cell Carcinoma of the Tongue: Analysis of Risk Factors, and Prognostic Value of the 8th Edition AJCC TNM Staging System. Frontiers in oncology. 2020;10. doi: 10.3389/fonc.2020.00984.

112. Farquhar DR, Tanner AM, Masood MM, Patel SR, Hackman TG, Olshan AF, et al. Oral tongue carcinoma among young patients: An analysis of risk factors and survival. Oral oncology. 2018;84:7-11. doi: 10.1016/j.oraloncology.2018.06.014.

113. Hilly O, Strenov Y, Rath-Wolfson L, Hod R, Shkedy Y, Mizrachi A, et al. The predictive value of dendritic cells in early squamous cell carcinoma of the tongue. Pathology Research and Practice. 2016;212(12):1138-43. doi: 10.1016/j.prp.2016.09.011.

114. Mair M, Nair D, Nair S, Malik A, Mishra A, Kannan S, et al. Comparison of tumor volume, thickness, and T classification as predictors of outcomes in surgically treated squamous cell carcinoma of the oral tongue. Head and Neck. 2018;40(8):1667-75. doi: 10.1002/hed.25161.

115. Ord RA, Isaiah A, Dyalram D, Lubek JE. Is Long-Term Follow-Up Mandatory for Stage I Oral Tongue Cancer? Journal of Oral and Maxillofacial Surgery. 2018;76(12):2676-83. doi: 10.1016/j.joms.2018.06.169.

116. Sakata J, Yamana K, Yoshida R, Matsuoka Y, Kawahara K, Arita H, et al. Tumor budding as a novel predictor of occult metastasis in cT2N0 tongue squamous cell carcinoma. Human Pathology. 2018;76:1-8. doi: 10.1016/j.humpath.2017.12.021.

117. Shen WR, Wang YP, Chang JYF, Yu SY, Chen HM, Chiang CP. Perineural invasion and expression of nerve growth factor can predict the progression and prognosis of oral tongue squamous cell carcinoma. Journal of Oral Pathology and Medicine. 2014;43(4):258-64. doi: 10.1111/jop.12133.

118. Tagliabue M, Gandini S, Maffini F, Navach V, Bruschini R, Giugliano G, et al. The role of the T-N tract in advanced stage tongue cancer. Head and Neck. 2019;41(8):2756-67. doi: 10.1002/hed.25761.

119. Tarsitano A, Del Corso G, Tardio ML, Marchetti C. Tumor Infiltration Depth as Predictor of Nodal Metastasis in Early Tongue Squamous Cell Carcinoma. Journal of Oral and Maxillofacial Surgery. 2016;74(3):523-7. doi: 10.1016/j.joms.2015.09.015.

120. Barrett AW, Pratt MK, Sassoon I, Bisase BS, Newman L, Tighe JV, et al. Perineural and lymphovascular invasion in squamous cell carcinoma of the tongue. Journal of Oral Pathology and Medicine. 2020. doi: 10.1111/jop.13104.

121. Daripally S, Peddi K. Polymorphic variants of drug-metabolizing enzymes alter the risk and survival of oral cancer patients. 3 Biotech. 2020;10(12):529. Epub 2020/11/21. doi: 10.1007/s13205-020-02526-5. PubMed PMID: 33214976; PubMed Central PMCID: PMCPMC7658280.

122. D'Cruz AK, Siddachari RC, Walvekar RR, Pantvaidya GH, Chaukar DA, Deshpande MS, et al. Elective neck dissection for the management of the N0 neck in early cancer of the oral tongue: need for a randomized controlled trial. Head & neck. 2009;31(5):618-24. Epub 2009/01/10. doi: 10.1002/hed.20988. PubMed PMID: 19132717.

123. Hicks J, Flaitz C. Mucoepidermoid carcinoma of salivary glands in children and adolescents: assessment of proliferation markers. Oral oncology. 2000;36(5):454-60. Epub 2000/08/30. doi: 10.1016/s1368-8375(00)00033-6. PubMed PMID: 10964053.

124. Huang Y, Lin C, Kao HK, Hung SY, Ko HJ, Huang YC, et al. Digital Image Analysis of CD8+ and CD3+ Tumor-Infiltrating Lymphocytes in Tongue Squamous Cell Carcinoma. Cancer management and research. 2020;12:8275-85. Epub 2020/09/29. doi: 10.2147/cmar.S255816. PubMed PMID: 32982423; PubMed Central PMCID: PMCPMC7490039.

125. Larson AR, Kemmer J, Formeister E, El-Sayed I, Ha P, George J, et al. Beyond Depth of Invasion: Adverse Pathologic Tumor Features in Early Oral Tongue Squamous Cell Carcinoma. The Laryngoscope. 2020;130(7):1715-20. Epub 2019/08/15. doi: 10.1002/lary.28241. PubMed PMID: 31411752.

126. Lee DY, Kang Y, Im NR, Kim B, Kwon TK, Jung KY, et al. Actin-Associated Gene Expression is Associated with Early Regional Metastasis of Tongue Cancer. The Laryngoscope. 2020. Epub 2020/11/26. doi: 10.1002/lary.29025. PubMed PMID: 33237593.

127. Liao CT, Lee LY, Hsueh C, Lin CY, Fan KH, Wang HM, et al. Clinical Outcomes in pT4 Tongue Carcinoma are Worse than in pT3 Disease: How Extrinsic Muscle Invasion Should be Considered? Annals of surgical oncology. 2017;24(9):2570-9. Epub 2017/06/04. doi: 10.1245/s10434-017-5906-3. PubMed PMID: 28577181.

128. Nagam SL, Katta S, Prasad VV. Gender specific association of TP53 polymorphisms (EX4 215G>C Arg72Pro, IVS3+40-41ins16, and IVS6+62G>A), with risk of oral cancer subtypes and overall survival of the patients. Molecular carcinogenesis. 2017;56(3):895-912. Epub 2016/08/18. doi: 10.1002/mc.22543. PubMed PMID: 27532290.

129. Pathak KA, Das AK, Agarwal R, Talole S, Deshpande MS, Chaturvedi P, et al. Selective neck dissection (I-III) for node negative and node positive necks. Oral oncology. 2006;42(8):837-41. Epub 2006/05/30. doi: 10.1016/j.oraloncology.2005.12.002. PubMed PMID: 16730221.

130. Sauter ER, Ridge JA, Gordon J, Eisenberg BL. p53 overexpression correlates with increased survival in patients with squamous carcinoma of the tongue base. American journal of surgery. 1992;164(6):651-3. Epub 1992/12/01. doi: 10.1016/s0002-9610(05)80727-5. PubMed PMID: 1463117.

131. Silva SD, Perez DE, Nishimoto IN, Alves FA, Pinto CA, Kowalski LP, et al. Fatty acid synthase expression in squamous cell carcinoma of the tongue: clinicopathological findings. Oral diseases. 2008;14(4):376-82. Epub 2008/04/16. doi: 10.1111/j.1601-0825.2007.01395.x. PubMed PMID: 18410580.

132. Theocharis S, Klijanienko J, Giaginis C, Rodriguez J, Jouffroy T, Girod A, et al. Expression of DNA repair proteins, MSH2, MLH1 and MGMT in mobile tongue squamous cell carcinoma: associations with clinicopathological parameters and patients' survival. Journal of oral pathology & medicine : official publication of the International Association of Oral Pathologists and the American Academy of Oral Pathology. 2011;40(3):218-26. Epub 2010/10/30. doi: 10.1111/j.1600-0714.2010.00945.x. PubMed PMID: 21029181.

133. Theocharis S, Kotta-Loizou I, Klijanienko J, Giaginis C, Alexandrou P, Dana E, et al. Extracellular signal-regulated kinase (ERK) expression and activation in mobile tongue squamous cell carcinoma: associations with clinicopathological parameters and patients survival. Tumour biology : the journal of the International Society for Oncodevelopmental Biology and Medicine. 2014;35(7):6455-65. Epub 2014/04/01. doi: 10.1007/s13277-014-1853-9. PubMed PMID: 24682903.

134. Wu CN, Chuang HC, Lin YT, Fang FM, Li SH, Chien CY. Prognosis of neutrophil-to-lymphocyte ratio in clinical early-stage tongue (cT1/T2N0) cancer. OncoTargets and therapy. 2017;10:3917-24. Epub 2017/08/24. doi: 10.2147/ott.S140800. PubMed PMID: 28831266; PubMed Central PMCID: PMCPMC5552147.

135. Yu SY, Wang YP, Chang JY, Shen WR, Chen HM, Chiang CP. Increased expression of MCM5 is significantly associated with aggressive progression and poor prognosis of oral squamous cell carcinoma. Journal of oral pathology & medicine : official publication of the International Association of Oral Pathologists and the American Academy of Oral Pathology. 2014;43(5):344-9. Epub 2013/11/20. doi: 10.1111/jop.12134. PubMed PMID: 24245508.

136. Yuen PW, Lam KY, Chan AC, Wei WI, Lam LK. Clinicopathological analysis of local spread of carcinoma of the tongue. American journal of surgery. 1998;175(3):242-4. Epub 1998/04/29. doi: 10.1016/s0002-9610(97)00282-1. PubMed PMID: 9560130.

137. Zanoni DK, Montero PH, Migliacci JC, Shah JP, Wong RJ, Ganly I, et al. Survival outcomes after treatment of cancer of the oral cavity (1985-2015). Oral oncology. 2019;90:115-21. Epub 2019/03/09. doi: 10.1016/j.oraloncology.2019.02.001. PubMed PMID: 30846169; PubMed Central PMCID: PMCPMC6417804.

138. Chakraborty S, M Patil V, Babu S, Muttath G, Thiagarajan SK. Locoregional recurrences after post-operative volumetric modulated arc radiotherapy (VMAT) in oral cavity cancers in a resource constrained setting: Experience and lessons learned. British Journal of Radiology. 2015;88(1048). doi: 10.1259/bjr.20140795.

139. Furukawa K, Kawasaki G, Naruse T, Umeda M. Prognostic significance of pretreatment lymphocyte-to-monocyte ratio in patients with tongue cancer. Anticancer research. 2019;39(1):405-12. doi: 10.21873/anticanres.13126.

140. Kim YI, Cheon GJ, Kang SY, Paeng JC, Kang KW, Lee DS, et al. Prognostic value of simultaneous 18F-FDG PET/MRI using a combination of metabolo-volumetric parameters and apparent diffusion coefficient in treated head and neck cancer. EJNMMI Research. 2018;8. doi: 10.1186/s13550-018-0357-9.

141. Narayan G, Jha R, Srikant P, Sinha S, Swarnalata G, Raju KVVN. Carcinoma of the tongue in renal transplant recipients: An unusual spectrum of de novo malignancy at a tertiary care center in India over a period of 26 years. Indian Journal of Nephrology. 2018;28(2):119-26. doi: 10.4103/ijn.IJN_354_16.

142. Patel KD, Barasiya YV, Patel JB, Patel PS. Apoptosis stimulating protein of p53 (ASPP) 1 and ASPP2 m-RNA expression in oral cancer. Archives of oral biology. 2020;119:104920. doi: 10.1016/j.archoralbio.2020.104920.

143. Tarsitano A, Asioli S, Morandi L, Monti V, Righi A, Morselli Labate AM, et al. Laminin-5 and insulin-like growth factor-II mRNA binding protein-3 (IMP3) expression in preoperative biopsy specimens from oral cancer patients: Their role in neural spread risk and survival stratification. Journal of Cranio-Maxillofacial Surgery. 2016;44(12):1896-902. doi: 10.1016/j.jcms.2016.07.012.

144. Zanoni DK, Migliacci JC, Xu B, Katabi N, Montero PH, Ganly I, et al. A Proposal to Redefine Close Surgical Margins in Squamous Cell Carcinoma of the Oral Tongue. JAMA otolaryngology-- head & neck surgery. 2017;143(6):555-60. doi: 10.1001/jamaoto.2016.4238. PubMed PMID: 28278337; PubMed Central PMCID: PMCPMC5473778.

145. Cracchiolo JR, Xu B, Migliacci JC, Katabi N, Pfister DG, Lee NY, et al. Patterns of recurrence in oral tongue cancer with perineural invasion. Head Neck. 2018;40(6):1287-95. Epub 2018/03/10. doi: 10.1002/hed.25110. PubMed PMID: 29522275; PubMed Central PMCID: PMCPMC5980694.

**Supplementary Table S3.** Quality of the included studies

| **Cochrane risk of bias assessment tool for randomized controlled trials** | | | | | | | | | | |
| --- | --- | --- | --- | --- | --- | --- | --- | --- | --- | --- |
| Study | 1 | | 2 | 3 | | | 4 | 5 | 6 | 7 |
| Yang, 2018 (45) | Low | | Unclear | Low | | | Low | Low | Low | Low |
| 1. Random sequence generation (selection bias); 2. Allocation concealment (selection bias); 3. Blinding of participants and personnel (performance bias); 4. Blinding of outcome assessment (detection bias); 5. Incomplete outcome data (attrition bias); 6. Selective reporting (reporting bias); 7. Other bias. | | | | | | | | | | |
| **NOS criteria for cohort studies** | | | | | | | | | | |
| Study | 1 | 2 | 3 | 4 | 5 | 6 | | 7 | 8 | Total quality scores |
| Choi, 2017 (46) | ★ | ★ | ★ | ★ | ★★ | ★ | | ★ | ★ | 9 |
| Cracchiolo, 2018 (26) | ★ | ★ | ★ | \ | ★★ | \ | | ★ | ★ | 7 |
| De Paz, 2019 (49) | ★ | ★ | ★ | ★ | ★★ | \ | | ★ | ★ | 8 |
| Durr, 2013 (27) | ★ | ★ | ★ | \ | ★★ | ★ | | ★ | ★ | 8 |
| Goodman, 2009 (48) | ★ | ★ | ★ | \ | ★★ | ★ | | ★ | ★ | 8 |
| Mascitti, 2020 (29) | ★ | ★ | ★ | \ | ★★ | ★ | | ★ | ★ | 8 |
| Ong, 2018 (30) | ★ | ★ | ★ | \ | ★★ | \ | | ★ | ★ | 7 |
| Sridharan, 2019 (50) | ★ | ★ | ★ | \ | ★★ | \ | | ★ | ★ | 7 |
| Subramaniam, 2020 (43) | ★ | ★ | ★ | ★ | ★★ | ★ | | ★ | ★ | 9 |
| 1. Representativeness of the exposed cohort; 2. Selection of the non-exposed cohort; 3. Ascertainment of exposure; 4. Demonstration that the outcome of interest was not present at the start of the study; 5. Comparability of cohorts based on the design or analysis; 6. Assessment of outcome; 7. Was follow-up long enough for outcomes to occur; 8. Adequacy of follow up of cohorts. | | | | | | | | | | |
| **Methodological items for non-randomized studies** | | | | | | | | | | |
| Study | 1 | 2 | 3 | 4 | 5 | 6 | | 7 | 8 | Total quality scores |
| Almangush, 2015 (25) | 1 | 1 | 0 | 1 | 1 | 1 | | 1 | 0 | 6 |
| Ling, 2013 (20) | 1 | 1 | 0 | 1 | 1 | 1 | | 1 | 0 | 6 |
| Marra, 2019 (28) | 1 | 1 | 0 | 1 | 1 | 1 | | 1 | 0 | 6 |
| Peng, 2014 (31) | 1 | 1 | 0 | 1 | 1 | 1 | | 1 | 0 | 6 |
| Sharma, 2019 (32) | 1 | 1 | 0 | 0 | 1 | 1 | | 1 | 0 | 5 |
| Thiagarajan, 2014 (44) | 1 | 1 | 0 | 1 | 1 | 1 | | 1 | 0 | 6 |
| Xu, 2020 (47) | 1 | 1 | 1 | 1 | 1 | 1 | | 1 | 1 | 8 |
| 1. A precisely stated aim; 2. Inclusion of consecutive patients; 3. Prospective collection of data; 4. Endpoints appropriate to the aim of the study; 5. Unbiased assessment of the study endpoint; 6. Follow-up period appropriate to the aim of the study; 7. Loss to follow-up <5%; 8. Prospective calculation of the study size. | | | | | | | | | | |

**Supplementary Table S4**. GRADE for each outcome

**Question**: Perineural invasion compared to Non-perineural invasion in oral tongue squamous cell carcinoma

| **Certainty assessment** | | | | | | | **№ of patients** | | **Effect** | | **Certainty** | **Importance** |
| --- | --- | --- | --- | --- | --- | --- | --- | --- | --- | --- | --- | --- |
| **№ of studies** | **Study design** | **Risk of bias** | **Inconsistency** | **Indirectness** | **Imprecision** | **Other considerations** | **Perineural invasion** | **Non-perineural invasion** | **Relative (95% CI)** | **Absolute (95% CI)** |  |  |
| **Recurrence** | | | | | | | | | | | | |
| 7 | non-randomised studies | not serious | not serious | not serious | not serious | none | 463 participants | 1196 participants | **HR 1.854** (1.412 to 2.435) [Recurrence] | **0 fewer per 1,000** (from 0 fewer to 0 fewer) | ⨁⨁⨁⨁ HIGH | CRITICAL |
|  |  |  |  |  |  |  | - | 100.0% |  | **0 fewer per 1,000** (from 0 fewer to 0 fewer) |  |  |
| **Overall survival** | | | | | | | | | | | | |
| 5 | non-randomised studies | not serious | not serious | not serious | not serious | none | 308 participants | 415 participants | **HR 1.944** (1.387 to 2.724) [Overall survival] | **-- per 1,000** (from -- to --) | ⨁⨁⨁⨁ HIGH | CRITICAL |
|  |  |  |  |  |  |  | - | 0.0% |  | **-- per 1,000** (from -- to --) |  |  |
| **Disease free survival** | | | | | | | | | | | | |
| 7 | non-randomised studies | not serious | not serious | not serious | not serious | all plausible residual confounding would suggest spurious effect, while no effect was observed | 458 participants | 999 participants | **HR 2.128** (1.532 to 2.955) [Disease free survival] | **-- per 1,000** (from -- to --) | ⨁⨁⨁⨁ HIGH | CRITICAL |
|  |  |  |  |  |  |  | - | 0.0% |  | **-- per 1,000** (from -- to --) |  |  |
| **Cancer specific survival** | | | | | | | | | | | | |
| 5 | non-randomised studies | not serious | not serious | not serious | not serious | all plausible residual confounding would suggest spurious effect, while no effect was observed | 322 participants | 884 participants | **HR 1.927** (1.402 to 2.650) [Cancer specific survival] | **-- per 1,000** (from -- to --) | ⨁⨁⨁⨁ HIGH | IMPORTANT |
|  |  |  |  |  |  |  | - | 0.0% |  | **-- per 1,000** (from -- to --) |  |  |

**CI:** Confidence interval; **HR:** Hazard Ratio
